# Interstitial boron-doped mesoporous semiconductor oxides for ultratransparent energy storage

**DOI:** 10.1038/s41467-020-20352-4

**Published:** 2021-01-19

**Authors:** Jian Zhi, Min Zhou, Zhen Zhang, Oliver Reiser, Fuqiang Huang

**Affiliations:** 1 State Key Laboratory of High-Performance Ceramics and Superfine Microstructure, Shanghai Institute of Ceramics, Chinese Academy of Sciences, Shanghai, P. R. China; 2 Institute of Organic Chemistry, University of Regensburg, Universitätsstr. 31, Regensburg, Germany; 3 Hefei National Laboratory for Physical Science at the Microscale, Department of Applied Chemistry, University of Science and Technology of China, Hefei, Anhui P. R. China; 4 SCNU-TUE Joint Lab of Device Integrated Responsive Materials (DIRM), South China Normal University, Guangzhou, China; 5 Beijing National Laboratory for Molecular Sciences and State Key Laboratory of Rare Earth Materials Chemistry and Applications, College of Chemistry and Molecular Engineering, Peking University, Beijing, P. R. China

**Keywords:** Electrochemistry, Supercapacitors, Electrochemistry

## Abstract

Realizing transparent and energy-dense supercapacitor is highly challenging, as there is a trade-off between energy storing capability and transparency in the active material film. We report here that interstitial boron-doped mesoporous semiconductor oxide shows exceptional electrochemical capacitance which rivals other pseudocapacitive materials, while maintaining its transparent characteristic. This improvement is credited to the robust redox reactions at interstitial boron-associated defects that transform inert semiconductor oxides into an electrochemically active material without affecting its transparency. By precisely tuning the level of doping, the pseudocapacitive reactivity of these materials is optimized, resulting in a volumetric capacitance up to 1172 F cm^−3^. Attributing to such efficient charge storage utilization on the active film, the fabricated transparent supercapacitor delivers a maximum areal energy density of 1.36 × 10^−3^ mWh cm^−2^ that is close to those of conventional pseudocapacitive materials, with nearly 100% capacitance retention after 15000 cycles and ultrahigh transparency (up to 85% transmittance at 550 nm). In addition, this device shows excellent durability and flexibility with multiple optional outputs, demonstrating the potential as a transparent energy supply in planar electronics.

## Abstract (web summary)

Realizing transparent supercapacitor is hindered by the trade-off between capacitance and transparency of the electrode material. Here, the authors report an interstitial doping strategy to boost the electrochemical activity of transparent conducting oxides while maintaining their optical feature.

## Introduction

Optical transparency and mechanical flexibility have become a new trend for the next generation of planar electronics that make possible emerging techniques, including electrochromic windows, electronic skin, wearable optoelectronics, and rolled-up displays^1^. To achieve these features, potential energy storage systems (EES) should be both flexible and transparent with ultrahigh areal energy density. As a critical power source, transparent and flexible supercapacitors (TFSCs) are promising EES devices in planar electronics, since they can deliver safe operation and high power density^2^. The areal energy density (*E*_areal_) in a TFSCs electrode is correlated to the volumetric capacitance (*C*_vol_), the thickness of active layer, and operating voltage (Δ*U*) as shown in Eq. (1)^3^:

1 $${{E}}_{{\mathrm{areal}}} = \frac{1}{2}{\mathrm{C}}_{{\mathrm{{vol}}}}l\Delta U^2$$

Accordingly, to achieve the highest *E*_areal_, all three variables (*C*_vol_, *l*, and Δ*U*) should be maximized. Increasing Δ*U* is straight-forward, as organic electrolytes permit a wider range of operating voltage (1–1.5 V) compared to traditional aqueous electrolytes (≤0.8 V)^4^. However, increasing *l* is challenging in TFSCs since the thickness of active material layer in the electrode should be kept below 100 nm to retain transparency^5^. As a result, for an ideal TFSCs electrode, *C*_vol_ of active materials should be high enough to overcome the trade-off between the transparency and energy-storing capability of the planar device.

One strategy to fabricate TFSCs is employing graphene and carbon nanotubes (CNTs) as electrode materials^6^. The graphene is intrinsically transparent in the multi-layer architecture, and the CNT films can achieve transparency in the porous structure. However, due to the electric double-layer capacitor (EDLC) charge storage mechanism and relatively low bulk density, the *C*_vol_ of such carbon-based materials is generally below 20 F cm^−3^, which results in a far from satisfactory *E*_areal_ below 0.008 µWh cm^−^^2^ (ref. ^7^). Besides carbon materials, there is also a vast number of research studies on supercapacitors focusing on pseudocapacitive materials, with charge stored not only through ion adsorption but also via near-surface redox reactions. Large gravimetric capacitance (*C*_gra_) over 1000 F g^−1^ was achieved in polyaniline^8^, MnO_2_ (ref. ^9^), RuO_2_ (ref. ^10^), and Co_3_O_4_ (ref. ^11^) based planar electrodes. Nevertheless, due to the non-transparent properties of these transition metal oxides, to tailor such materials into transparent electrodes remains a formidable challenge.

Recently, advances in electrode design have allowed the fabrication of TFSCs with some transparency. For example, Zhang et al.^12^ produced a ruthenium oxide/poly(3,4-ethylenedioxythiophene): poly(styrene-4-sulfonate), (RuO_2_/PEDOT:PSS) hybrid TFSC, which reaches a 80% transparency with an *E*_areal_ of 0.015 µWh cm^−^^2^. In another report, Sheng and co-workers synthesized ultrathin wrinkled Co(OH)_2_ nanosheets vertically grown on silver nanowires (Ag NWs). The obtained TFSCs achieved 50% transparency and an *E*_areal_ of 0.04 μWh cm^−2^, with excellent mechanical flexibility^13^. However, despite some progress, it is still very difficult, if not impossible, to enable efficient energy storage in TFSCs without sacrificing the transparency of the electrode. As a result, further exploration of other electrode materials with superb *C*_vol_ and optical properties is highly desirable to make TFSCs feasible, which moreover should be easily fabricated from inexpensive materials to allow for large-scale application.

Transparent conducting oxides (TCOs) derived from semiconductor oxides, such as fluorine-doped SnO_2_ (FTO), indium-doped SnO_2_ (ITO) and boron-doped ZnO (BZO), play important roles as transparent conductive materials in commercial applications such as display and lighting devices^14^. In typical FTO, ITO, and BZO, one metal site from semiconductor oxide is substituted with one dopant, which provides one charge carrier for conduction between the lattices. However, due to the less accessible charge storage sites^15^, TCOs are generally considered as electrochemical inert materials with low *C*_vol_ (normally <10 F cm^−3^) that is not feasible in TFSCs.

In this work, we report a series of TCOs, interstitial boron (B)-doped mesoporous semiconductor oxides (SnO_2−*x*_B_*y*_, ZnO_1−*x*_B_*y*_, or In_2_O_3−*x*_B_*y*_), that have impressive electrochemical redox properties, while maintaining their transparent characteristics. Such “interstitial boron” not only boosts the carrier densities of the doped materials, but also adopts diverse coordination environment with abundant oxygen vacancies, which provides accessible sites for OH^−^ intercalation and leads to robust redox reactions at boron-associated defects. By precisely controlling the doping concentration of boron, the pseudo-capacitance of semiconductor oxides can be finely tuned, which allows the electrode to reach a *C*_vol_ of up to 1172 F cm^−3^ that rivals those of other pseudocapacitive materials. Attributing to such efficient charge storage utilization on the active film, the assembled TFSCs employing two identical highly transparent (up to 91%) electrodes fabricated through a scalable aerosol-jet spraying technology, delivered a *E*_areal_ of up to 1.36 × 10^−3^ mWh cm^−2^ at an optical transmittance as high as 85%, with nearly 100% capacitance retention after 15,000 cycles. Moreover, these TFSCs shows superb durability and flexibility with multiple optional outputs, indicating the promise as efficient power source for planar electronics.

## Results

**Fig. 1:**
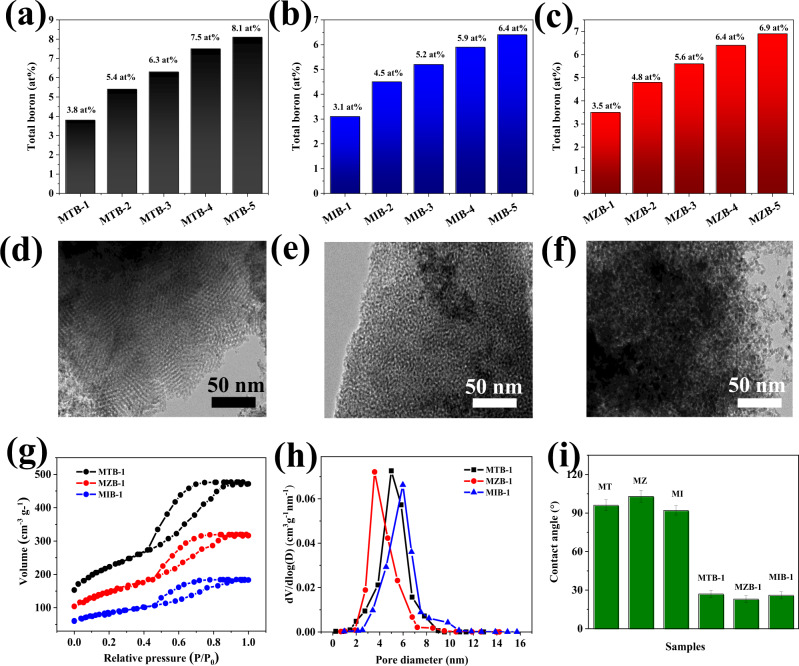
Structure of boron-doped mesoporous semiconductor oxides. **a**–**c** Summary of boron-doping level in MTB, MZB, and MIB series samples. **d**–**f** High-resolution transmission electron microscopy (HR-TEM) of MTB-1 (**a**), MZB-1 (**b**), and MIB-1 (**c**). **g**, **h** N_2_ adsorption−desorption isotherms and corresponding pore size distribution of mesoporous MTB-1, MZB-1, and MIB-1. **i** Contact angles of MTB-1, MZB-1, MIB-1 and the undoped samples (MT, MZ, and MI).

Interstitial boron-doped mesoporous semiconductor oxides were prepared (see the “Methods” section for details), starting with mesoporous semiconductor oxides synthesized from a typical evaporation-induced self-assembly (EISA) process. By changing the concentration of boric acid in the initial sol for EISA, a set of samples with various boron-doping concentrations was obtained (Fig. 1a–c and Table S1). The microstructure of interstitial boron-doped mesoporous SnO_2_, ZnO, and In_2_O_3_ samples with 3.8, 3.5, and 3.1 atomic percent of boron (denoted as MTB-1, MZB-1, and MIB-1, respectively) was firstly examined by transmission electron microscopy (TEM). MTB-1 exhibits a well-ordered structure as evidenced from the presence of the straight lattice edge (Fig. 1d), while MZB-1 and MIB-1 show sponge-like disordered structures (Fig. 1e, f). All the samples display a similar microstructure as the undoped ones (Supplementary Fig. 1), indicating the largely preserved mesoporous textures during doping. Attributing to the abundance of mesopores as confirmed from the typical type-IV isotherm in nitrogen adsorption–desorption analysis (Fig. 1g), MTB-1, MZB-1, and MIB-1 have a high surface area of 472, 274, and 156 m^2^ g^−1^ (Table S1), respectively, with a narrow pore size distribution centered from 4 to 8 nm (Fig. 1h). The boron doping also profoundly altered the wetting properties as well. Whereas undoped mesoporous SnO_2_, ZnO, and In_2_O_3_ (MT, MZ, and MI) are hydrophobic, MTB-1, MZB-1, and MIB-1 are hydrophilic (Fig. 1i). This is consistent with the zeta potential: boron-doped samples are more nucleophilic (zeta potential = −13.2, 16.7, and 24.4 mV in MTB-1, MZB-1, and MIB-1, respectively) than mesoporous semiconductor oxides (−3.6, −5.9, and 7.3 mV in MT, MZ, and MI, respectively) because of lone-pair B 2*p* electrons. This improved wettability of boron-doped samples is generally conducive to the energy storage performance.

**Fig. 2:**
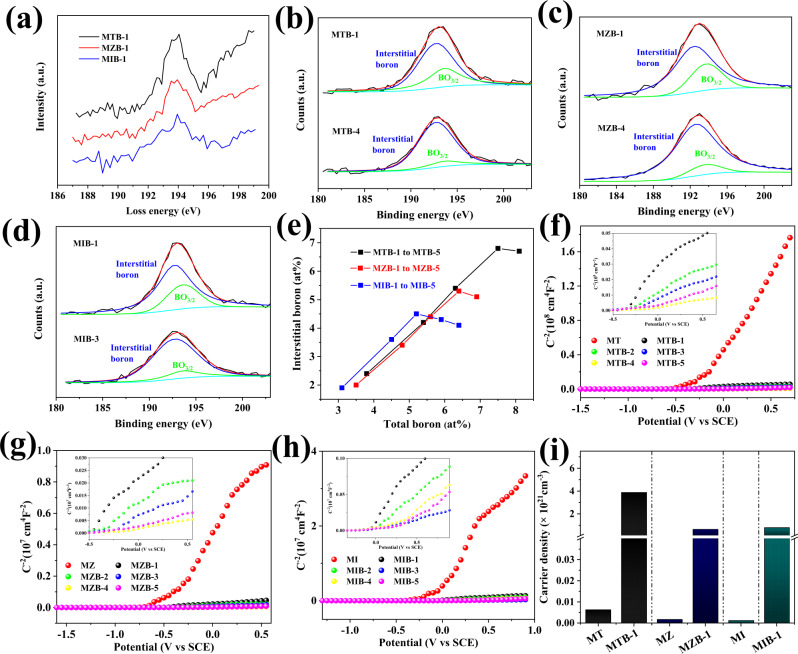
Spectroscopic and electronic characterization. **a** Electron energy loss spectroscopy (EELS) spectra from MTB-1, MZB-1, and MIB-1. **b**–**d** High-resolution XPS spectra for B 1*s* of MTB-1 and MTB-5, MZB-1 and MZB-5, and MIB-1 and MIB-5. **e** Interstitial boron (at%) as a function of total doped boron determined from XPS analysis in MTB-1 to MTB-5, MZB-1 to MZB-5, and MIB-1 to MIB-5. **f**–**h** Mott−Schottky plots for MTB-1 to MTB-5, MZB-1 to MZB-5, MIB-1 to MIB-5, and the undoped samples (MT, MZ, and MI). **i** Calculated carrier densities of MTB-1, MZB-1, MIB-1 and their corresponding pristine samples MT, MZ, and MI.

Spectroscopic studies identified the chemical state of the boron dopant incorporated into the lattice of semiconductor oxides. In electron energy loss spectrum (EELS; Fig. 2a), the peak at 193.8 eV in MTB-1, MZB-1, and MIB-1 corresponding to *sp*^2^ hybridized boron matches well with those of B_2_O_3_ and BO_3_ (ref. ^16^). In addition, the Sn/B, Zn/B, and In/B bonding of the doped samples quantified by X-ray photoelectron spectroscopy (XPS; Fig. 2b–d) revealed the following: (i) deconvoluted boron 1*s* XPS shows two peaks at 192.2 and 193.1 eV, corresponding to interstitial boron and surface BO_3/2_ species, respectively^17^. (ii) As the boron content increases from 3.8, 3.5, and 3.1 at% in MTB-1, MZB-1, and MIB-1, respectively, to 7.5, 6.4, and 5.2 at% in MTB-4, MZB-4, and MIB-3, respectively (Fig. 2e and Table S1), interstitial boron instead of surface BO_3/2_ becomes more abundant. As evidenced from O1*s* XPS spectrum (Supplementary Fig. 2), there are abundant interstitial oxygen O^2−^ in the lattice of MT, MZ, and MI, which is mainly derived from the trapped oxygen in the mesopores. To maintain the overall charge neutrality in the lattice, such negative charged interstitial oxygens prompt the positive charged boron to occupy the interstitial site, enabling an interstitial doping^18^. Interstitial boron with much smaller ionic radius than those of Sn^4+^, Zn^2+^, and In^3+^ adopts diverse coordination environment, and generally forms tetrahedron BO_4_ or triangle BO_3_ in the mesoporous samples without the need of excess oxygen. Therefore, abundant oxygen vacancies form around the doping sites, which leads to apparent symmetrical signals at *g* = 2.002 in the electron paramagnetic resonance spectra of the doped samples (Supplementary Fig. 3). In contrast, no signals were detected in MT, MZ, and MI, indicating free of paramagnetic species existing in undoped samples. (iii) Continuously increasing B content to 8.1, 6.9, and 5.9 at% as found in MTB-5, MZB-5, and MIB-4 leads to new characteristic peaks at 190.2, 190.6, and 190.35 eV (Supplementary Fig. 4), which can be attributed to the substitution boron with Sn, Zn, and In ions, respectively^19^.

The crystal structure of interstitial boron-doped (MTB-1, MZB-1, and MIB-1) and pristine (MT, MZ, and MI) semiconductor oxides was further revealed by X-ray diffraction (XRD) spectroscopy. All peaks are readily indexed to SnO_2_ with six-coordinate octahedron SnO_6_ (Supplementary Fig. 5, JCPDS no. 41-1445), ZnO with four coordinate tetrahedron ZnO_4_ (Supplementary Fig. 6, JCPDS no. 36-1451), and In_2_O_3_ with six-coordinate octahedron InO_6_ (Supplementary Fig. 7, JCPDS no. 06-0416). Extracted from Le Bail refinement tooled with General Structure Analysis System, the crystallite size, and unit cell volume of MTB-1, MZB-1, and MIB-1 were shown to be larger than the ones of pristine samples (Table S2), which is mainly due to the interstitial incorporation of boron with an ionic radium of 0.3 Å in the lattice^19^. It should be noted that such interstitial occupation of boron differs from the boron-doped SnO_2_, ZnO, and In_2_O_3_ via the low-pressure chemical vapor deposition (denoted as TB-LPCVD, ZB-LPCVD, and IB-LPCVD, respectively) that is widely used to produce commercial TCOs^20^. In these LPCVD samples, boron was found to occupy the metal-substitutional position. Such substitutional doping leads to the contraction of the lattice parameter in the unit cell (Supplementary Figs. 8–10 and Table S2), which is due to the much smaller radius of the substitutional boron dopant than the metallic atom.

To study the impact of interstitial boron doping on the electronic properties of SnO_2_, ZnO, and In_2_O_3_ samples, Mott–Schottky analysis of impedance was conducted. Positive slopes indicate that all samples are n-type semiconductor and boron doping has no influence on transition in the type of semiconductor (Fig. 2f–h). The carrier densities of semiconductor materials can be calculated employing the Mott–Schottky equation:

2 $$N_{\mathrm{d}} = \left( {\frac{2}{{\epsilon _0\varepsilon \varepsilon _0}}} \right)\left[ {\frac{{{\mathrm{d}}\left( {\frac{1}{{c^2}}} \right)}}{{{\mathrm{d}}v}}} \right]^{ - 1}$$

where ${\it{\epsilon }}_0$, $\varepsilon$, $\varepsilon _0$, $N_{\mathrm{d}}$, $\frac{{{\mathrm{d}}(\frac{1}{{c^2}})}}{{{\mathrm{d}}v}}$ represent the electron charge, the dielectric constant of the sample, the permittivity of vacuum, the carrier density, and the straight slope, respectively^21^. Based on this equation, the carrier densities of MTB-1, MZB-1, and MIB-1 is 3.87 × 10^21^, 6.28 × 10^20^, 7.79 × 10^20^ cm^−3^, respectively, which is at least three orders of magnitude larger than the pristine MT, MZ, and MI samples (Fig. 2i). The significantly increased carrier densities in the boron-doped samples is mainly due to the formation of oxygen vacancies in the interstitial site of the lattice, which releases free electrons and greatly alter the resistivity of the doped samples. Whereas pristine MT, MZ, and MI are clearly semiconductors, MTB-1, MZB-1, and MIB-1 exhibit much lower resistance under room temperature with weaker dependence on temperature (Supplementary Fig. 11). These trends are in close agreement with results reported for FTO, ITO, and BZO, suggesting the similar fast-conducting feature as conventional TCOs. By increasing the doping concentration, the carrier densities of the samples can be further boosted, which results in the maximum values of 2.32 × 10^22^, 9.87 × 10^21^, and 1.02 × 10^21^ cm^−3^ in MTB-4, MZB-4, and MIB-3, respectively. However, there is a saturation point in the carrier densities of boron-doped samples. When the doping concentration was further increased (MTB-5, MZB-5, and MIB-4), the carrier density was reduced accordingly (Fig. 2f–h). The increased incorporation of substitutional boron (Supplementary Fig. 4) would trap free electrons and leads to reduction of the free electron concentration ^22^.

**Fig. 3:**
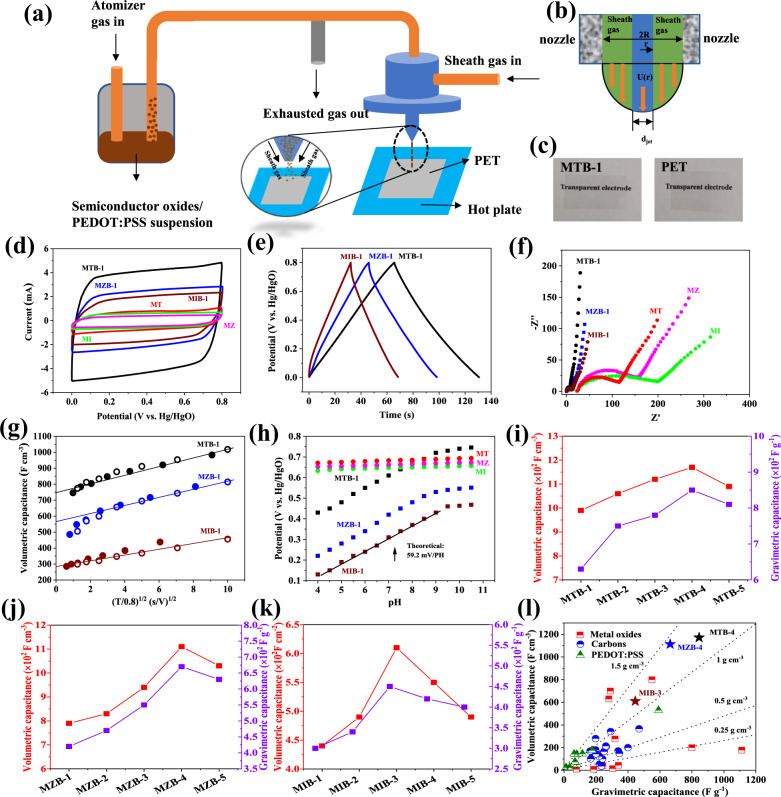
Fabrication and electrochemical evaluation of transparent electrodes. **a**, **b** Schematic representation of aerosol-jet spraying technology and flow profile inside a nozzle^23^. **c** Photographs of the transparent electrode. **d**–**f** Cyclic voltammetry (0.05 V s^−1^, **d**), galvanostatic charge/discharge (0.1 mA cm^−2^, **e**), and Nyquist plots (**f**) for B-doped and undoped samples. **g** Capacity along with square root of half-cycle time for B-doped samples from CV (solid circles, 0.01–0.64 V s^−1^) and CC data (open circles, 0.1–6.4 mA cm^−2^). **h** Tafel plots of potential with pH value at steady-state current density of 10 mA cm^−2^ for B-doped and undoped samples. **i**–**k** Optimization of *C*_vol_ and *C*_gra_ in B-doped samples at 0.1 mA cm^−2^. **l** *C*_vol_ and *C*_gra_ of MTB-4, MZB-4, MIB-3 compared with carbon^24–41^, metal oxides^8,42–52^, and PEDOT:PSS^53–61^.

In this study, we have used the aerosol-jet spraying strategy to prepare transparent supercapacitor electrodes employing polyethylene terephthalate (PET) as transparent substrate. According to this method, interstitial boron-doped and pristine samples are aerosolized with poly(3,4-ethylenedioxythiophene):poly(styrene-4-sulfonate), (PEDOT:PSS, 75 wt% of oxides) and entrained in a gas stream (Fig. 3a). The gas is transferred to the head of deposition and then compressed with a coaxial sheath gas flow^23^, as shown in Fig. 3b (*d*_jet_: the aerosol-jet diameter; *U*(*r*): the effective aerosol stream flow profile; *R*: nozzle radius). Such aerosol-jet technology is able to coat sols on conformal substrates, demonstrating a scalable technology for depositing films on various devices such as transistors, strain gauges, and solar cells^62,63^. By adjusting the nozzle size and sheath gas/aerosol stream flow rate (Table S3), we optimized the aerolsol jet diameter (*d*_jet_) and produced a series of highly transparent (>88% at 550 nm; Fig. 3c) and conductive (<100 Ω/square) thin films based on various semiconductor oxides (Table S3). We found that boron doping on semiconductor oxides plays a key role on the electrical properties of the deposited films. For example, the sheet resistance of interstitial boron-doped mesoporous SnO_2_ decreases along with the increase of the doping concentration and reaches a steady value of 25 Ω/square at a boron content of 7.5 at% (MTB-4). On the other hand, the transmittance of boron-doped samples is slightly reduced along with the increase of the doping concentration. This is likely due to the excitation of electrons from the energy levels that resulted from the boron atoms, to the conduction band edge, which results in some visible light absorption^64^. However, the films are still highly transparent even in the most heavily doped samples (MTB-5, MZB-5, and MIB-5), which may be due to the expansion of unit cell volume through interstitial boron doping as confirmed from XRD analysis (Supplementary Figs. 5–7).

To obtain the film thickness that is critical in calculating *C*_vol_, Eq. (3) was employed ^65^:

3 $$T^{ - 0.5} - 1 = 188.5\sigma _{\mathrm{{op}}}(t)$$

where *t* is the thickness. A series opaque film on glass slides was prepared additionally and the corresponding thickness and transmittance were measured. As shown in Supplementary Fig. 12, the fitted slope equals the optical conductivity of $\sigma _{{\mathrm{{op}}}}$, and the deposited film thickness could be calculated by using Eq. (3). To verify the accuracy of the extrapolation, we further employed surface profiler Veeco Dektak 8 stylus to directly measure the thickness of all the transparent films as listed in Table S3. To minimize the testing error, three measurement points were chosen, and the film thickness was obtained by averaging the tested value of each point. Supplementary Figure 13 shows the representative profilometry results of MIT-4 film at three different testing points. The average thickness of MIT-4 is 118 nm, which is close to the value obtained from Eq. (3) (101 nm, Table S3). Similarly, other films also show a profilometry thickness that is consistent to the extrapolated data (in the brackets under the “Thickness” title in Table S3). As a result, we believe that such extrapolation method in estimating film thickness is reliable and accurate.

The electrochemical properties of the semiconductor oxide-based transparent films were tested in a three-electrode cell employing Pt as a counter electrode and Hg/HgO as a reference electrode. In 1 M KOH aqueous electrolyte, the significant improvement of MTB-1, MZB-1, and MIB-1 over MT, MZ, and MI is evident from cyclic voltammograms (CV; Fig. 3d), which show a significant larger current response at a scan rate of 0.05 V s^−^^1^. The CV curves of MTB-1, MZB-1, and MIB-1 electrode is rectangular, which is typical for capacitive behavior. The absence of pseudocapacitive peaks in the CV profile of transparent electrode is possibly due to the band structures of semiconductor oxides. As a result of boron doping, the apparent band gap of the semiconductor is increased, and the electrons populate in the conduction band minimum^66^. This phenomenon, called as “Moss–Burstein effect”^67^, provides sufficient electrons for fast and successive pseudocapacitive reactions between interstitial boron and OH^−^ over the whole voltage window, which leads to an EDLC-like CV shape that is similar to MnO_2_ and RuO_2_ (ref. ^68^). Consistent with the CV results, the galvanostatic charge/discharge tests (CC) in MTB-1, MZB-1, and MIB-1 show symmetric features with a linear slope (Fig. 3e). By knowing the film thickness as determined from Eq. (3), a *C*_vol_ as high as 990 F cm^−3^ at a current density of 0.1 mA cm^−2^ was obtained for MTB-1, versus 788 F cm^−3^ for MZB-1 and 439 F cm^−3^ for MIB-1 (Supplementary Fig. 14). Under different current densities, boron-doped samples continuously provide a high capacitance, achieving 796, 550, and 285 F cm^−3^ at 3.2 mA cm^−2^ in MTB-1, MZB-1, and MIB-1, respectively. It is well known that in some cases PEDOT:PSS itself also shows some capacitance^12^. To exclude the impact of PEDOT:PSS on the overall performance of the electrode, we prepared non-transparent MTB-4, MZB-4, and MIB-3 electrodes, employing acetylene black (AB) as a conductive agent and graphite foil as a current collector. Supplementary Figure 15a shows the galvanostatic charge/discharge profiles of MTB-4/AB, MZB-4/AB, MIB-3/AB electrodes at 0.4 mA cm^−2^. Under the same alkaline electrolyte (1 M KOH) that was used for transparent electrode, all three samples exhibit symmetric sloping curves, indicating highly capacitive behaviors. Derived from galvanostatic charge/discharge profiles, MTB-4/AB, MZB-4/AB, and MIB-3/AB based electrodes show a *C*_gra_ of 713, 538, and 385 F g^−1^ at 0.4 mA cm^−2^, respectively (Supplementary Fig. 15d), which is consistent to the results on their transparent counterparts (Fig. 3i–k, also shown in Supplementary Fig. 15d for comparison). Similar trends can be observed from MTB-4, MZB-4, and MIB-3 based electrode using nanotubes (CNT; Supplementary Fig. 15b, e) and graphite particles (KS-6; Supplementary Fig. 15c, f) as a conductive agent. It should be noted that the *C*_gra_ of AB, CNT, and KS-6 is only 30–50 F g^−1^ due to their EDLC mechanism (Supplementary Fig. 15d–f). Therefore, the extra capacitance of such non-transparent electrode is undoubtedly derived from MTB-4, MZB-4, and MIB-3, which also contributes to the high capacitance of MTB-4/PEDOT:PSS, MZB-4/PEDOT:PSS, and MIB-3/PEDOT:PSS based electrode. Moreover, the electrochemical behavior of PEDOT:PSS is based on their fast protonation/deprotonation reactions^69–71^. Note that the electrolyte employed here is 1 M KOH or PVA/KOH gel. As a result, there is no sufficient protons to account for the high pseudo-capacitance of PEDOT:PSS. Based on galvanostatic charge/discharge profile even at a low current density (0.01 mA cm^−2^; Supplementary Fig. 16), pristine PEDOT:PSS electrode shows a volumetric capacitance of only 12 F cm^−3^. Obviously, under alkaline electrolyte, the contribution of PEDOT:PSS to the total capacitance of transparent electrode in this work is negligible. Electrochemical impedance spectroscopy (Fig. 3f) found MTB-1, MZB-1, and MIB-1 to have much lower equivalent series and charge transfer resistance (*R*_es_ and *R*_ct_) than in MT, MZ, and MI. This may be attributed to the better wetting and much lower resistivities of boron-doped samples as confirmed in Fig. 1f and Fig. 2i, respectively. In addition, the slope of the impedance plot from boron-doped samples was much steeper than the slope from pristine samples, which further confirms the capacitive behavior at fast rates.

The CV and CC tests of MTB-1, MZB-1, and MIB-1 are in excellent agreement with each other under the same half-cycle time *T* in Fig. 3g, which further provides valuable information into the kinetics of charge/discharge. In general, the capacitance *C* includes a rate-independent parameter *k*_1_ and a diffusion controlled parameter *k*_2_ determined by the scanning rate, *v* = U/T, where *U* represents the voltage window (0.8 V in this case). This may be expressed as^72^

4 $$C = k_1 + k_2v^{ - 1/2}$$

In Fig. 3g, the $k_2v^{ - 1/2}$ term stands for the long-*T*, which extrapolate to *k*_1_ at the $(\frac{T}{{0.8}})^{1/2}$ = $v^{ - 1/2}$ = 0 intercept. Apparently, *k*_1_ dominates in boron-doped samples, reaching 750, 566, and 283 F cm^−3^ in MTB-1, MZB-1, and MIB-1, respectively. Dominance of rate-independent capacitances is common for carbon-based electrode derived from EDLC mechanism, but it also holds in redox reactions of semiconductor oxides described in this study because (i) interstitial boron-doped samples are high-surface-area materials with high concentration of free electrons (Fig. 2e–g) and (ii) interstitial boron-doped samples are mesoporous (Fig. 1c, d) and hydrophilic (Fig. 1f). As a result, they permit facile reactions both inside and outside the mesoporous channels, as well as across the walls of the channels. Moreover, according to the density functional theory (DFT) calculations, SnO_2_, ZnO, and In_2_O_3_ with interstitial boron atom present an appropriate OH^−^ intercalation energy (*E*_int_) of −6.323, −4.337, and −8.381 eV, respectively (Supplementary Fig. 17), which is in sharp contrast to those in pristine SnO_2_ (−2.448 eV), ZnO (−1.994 eV), and In_2_O_3_ (−4.581 eV). It is mainly due to the diverse coordination environment around the doping site of interstitial boron that forms abundant binding sites towards OH^−^. In addition, oxygen vacancies derived from the interstitial doping can also attract OH^−^ ions, which further decrease the binding barriers between B and OH^−^^73^. This indicates that as compared to undoped samples, the interstitial boron doping provides more stable storage sites for OH^−^, which is significant to achieve pseudocapacitive redox reaction between interstitial B and OH^−^.

The results based on near-equilibrium electrochemical characterizations encouraged us to build the Tafel plots in Fig. 3h and Supplementary Fig. 18 to demonstrate the kinetics of faradaic reactions and study the fundamental difference between boron doped and pristine semiconductor oxides. For MTB-1, MZB-1, and MIB-1, the potential needed to sustain a constant current density of 50 µA cm^−2^ in aqueous electrolyte with pH value from 4 to 10.5 (Fig. 3h) is close to the 2.3 × RT/F Tafel line with a slope of 59.2 mV/pH. Besides, testing the potential needed in various current densities (Supplementary Fig. 18) in 1 M KOH electrolyte provides again a slope in close coincidence with 2.3 × RT/F. As all the Tafel lines suggest a reaction that involves one electron, such pH dependence must derive from the concurrent intercalation of one OH^−^ and deintercalation of one electron. In comparison, for undoped samples (MT, MZ, and MI), the curve is quite flat (Fig. 3h), indicating little redox activity. As a result, we believe that the improved pseudo-capacitance in boron-doped semiconductor oxides shares a similar redox reaction, i.e. that each interstitial boron atom can incorporate an OH^−^, and meanwhile one electron is released from boron to account for the pseudocapacitive reactivity:

5 $${\mathrm{SnO}}_{2 - x}{\mathrm{B}}_y + {\mathrm{OH}}^ - \to {\mathrm{SnO}}_{2 - x}{\mathrm{B}}_y:{\mathrm{OH}} + {{e}}^ -$$

6 $${\mathrm{ZnO}}_{1 - x}{\mathrm{B}}_y + {\mathrm{OH}}^ - \to {\mathrm{ZnO}}_{1 - x}{\mathrm{B}}_y:{\mathrm{OH}} + {{e}}^ -$$

7 $${\mathrm{In}}_2{\mathrm{O}}_{3 - x}{\mathrm{B}}_y + {\mathrm{OH}}^ - \to {\mathrm{In}}_2{\mathrm{O}}_{3 - x}{\mathrm{B}}_y:{\mathrm{OH}} + {{e}}^ -$$

For LPCVD samples with substitutional boron doping, there is no pH dependence in Tafel plots (Supplementary Fig. 19). Therefore, we conclude that such redox reaction only occurs between interstitial boron and OH^−^. DFT calculations also shows that SnO_2_, ZnO, and In_2_O_3_ with substitutional boron atom present an *E*_int-OH_^−^ of −1.091, −1.339, and −2.118 eV, respectively. These values are even higher than the *E*_int-OH_^−^ in pristine SnO_2_, ZnO, and In_2_O_3_ (Supplementary Fig. 17), energetically suggesting the weak interaction between substitutional boron and OH^−^. As a result, in samples with substitutional boron, there are no OH^−^storage sites to realize B–OH^−^ redox reaction, which leads to the poor pseudocapacitive activity.

It should be noted that PEDOT:PSS/metal oxide-based transparent supercapacitors have already been studied^12^. There are also a few reports on the boron-doped MnO_2_^74^ and NiO^75^ as active materials for non-transparent supercapacitors. However, the metal oxides employed in these works are traditional redox capacitive material with high theoretical pseudo-capacitance. Either PEDOT:PSS or boron atom only acted as additive/dopant to increase the electrical conductivities of electrode materials, which did not actually change the energy storage mechanisms in metal oxides. In contrast, our work is conceptually different. SnO_2_, ZnO, and In_2_O_3_ are well-known TCOs for flat panel displays and thin film solar cells^76^. Nevertheless, due to the less accessible charge storage sites, TCOs are generally considered as electrochemically inert materials with much lower specific capacitance than transition metal oxides such as RuO_2_, NiO, or MnO_2_. In this work, our boron-doping approach in the interstitial site simultaneously boosts the carrier densities of the doped materials and adopts diverse coordination environment with abundant oxygen vacancies. Clearly, such interstitial boron doping completely alter the electrochemical activity of TCOs. As confirmed from pH-dependent Tafel plots for interstitial doped and undoped samples in Fig. 3h, and for substitutional doped samples in Supplementary Fig. 19, the increased pseudo-capacitance of TCOs stems from the robust redox reaction between interstitial boron and OH^−^. Compared to previous studies in pseudocapacitive materials that mainly derived from the intrinsic redox activities of metal oxides, such “interstitial doped boron” involved redox reaction in accounting for the pseudo-capacitance indeed shows a conceptual innovation in energy storage mechanism.

The optimal active materials for TFSCs requires its *C*_vol_ to be maximized to overcome the trade-off between the transparency and energy storing capability. Figure 3i–k illustrates the impact of boron doping on the capacitance of semiconductor oxides, exhibiting that *C*_vol_ can be greatly increased with the enhancing of boron-doping concentration. However, such capacitance enhancement is not monotonical with the increase of the doping level, and reaches a maximum value of 1172, 1113, and 610 F cm^−3^ in MTB-4, MZB-4, and MIB-3 at a boron content of 7.5, 6.4, and 5.2 at%, respectively. This trend is exactly the same as the correlation between carrier densities and doping concentration (Fig. 2e–g), thus strongly implying that the redox reaction is connected to the boron doping, and the free electrons from oxygen vacancies play a key role in the capacitance of the samples. Such optimized doping implies the most efficient charge storage behavior in planar electrode, which enables *C*_vol_ and *C*_gra_ of boron-doped semiconductor oxides to simultaneously reach a superior level compared with all known carbon^24–41^, metal oxides^8,42–52^, and PEDOT:PSS^53–61^ based electrodes under comparable conditions (Fig. 3l). It should be noted that in the MTB-4, MZB-4 and MIB-3 samples with the highest *C*_gra_ at 0.1 mA cm^−2^, the doped boron may store an additional faradic charge of 678, 571, and 465 F g^−1^, respectively. This is sufficient to account for the storage diffidence (649, 524, and 363 F g^−1^ to the *C*_gra_ at 0.1 mA cm^−2^ of MT MZ, and MI, respectively; Supplementary Fig. 20) between doped and pristine samples. The proposed B–OH^−^ mechanism dictates that alkaline electrolyte is more favorable for such reactions. This was further confirmed for MTB-4 in a CC test under 1 M H_2_SO_4_ electrolyte: It shows a much smaller *C*_vol_ (498 F cm^−3^; Supplementary Fig. 21) than in 1 M KOH electrolyte (1172 F cm^−3^) at 0.1 mA cm^−2^. In comparison, the undoped sample MT, which does not involve any B–OH^−^ redox reaction, has very similar *C*_vol_ in both basic (1 M KOH) and acidic electrolytes (1 M H_2_SO_4_, Supplementary Fig. 22). These data lend further support to the proposed B–OH^−^ redox reaction that makes MTB, MZB, or MIB a superior material for supercapacitors.

**Fig. 4:**
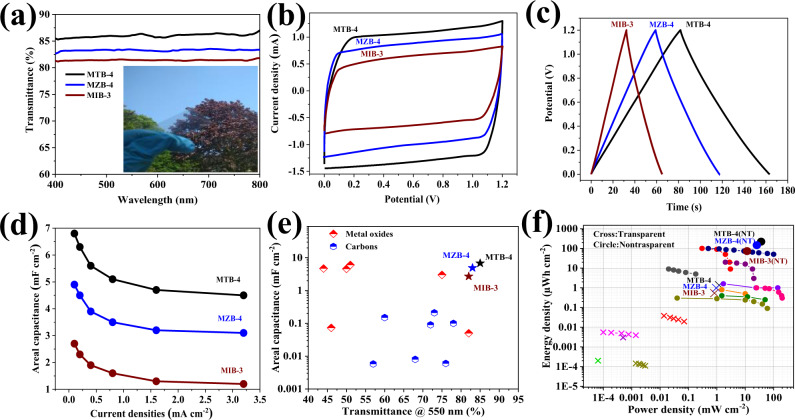
Optical and electrochemical evaluation of TFSCs. **a** Transmittance spectra of TFSCs based on MTB-4, MZB-4, and MIB-3 electrodes. Inset: photograph of TFSCs based on MTB-4 electrode. **b** Cyclic voltammetry (CV test at 0.05 V s^−1^) for MTB-4, MZB-4, and MIB-3 based TFSCs. **c** Galvanostatic charge/discharge (CC test at 0.1 mA cm^−2^) for MTB-4, MZB-4, and MIB-3 based TFSCs. **d** Areal capacitance of MTB-4, MZB-4, and MIB-3 based TFSCs at different current densities. **e** Comparing the areal capacitance and transparency (at 550 nm) of our TFSCs with recent carbon^7,65,77–81^ or metal oxide^82–87^ based transparent supercapacitors. **f** Ragone plots of MTB-4, MZB-4, and MIB-3 based TFSCs and non-transparent devices (MTB-4(NT), MZB-4(NT), and MIB-3(NT)) compared with previously reported values in state-of-the-art transparent (cross symbol)^7,65,77–79^ and non-transparent (circle symbol)^42,88–94^ planar supercapacitors.

The maximized *C*_vol_ in interstitial boron-doped semiconductor oxides through doping-level tuning encouraged us to fabricate a TFSCs that simultaneously possess high capacity and transparency, by assembling two identical transparent electrodes with a polyvinyl alcohol (PVA)-KOH gel electrolyte. The fabricated supercapacitor device employing MTB-4, MIB-4, and MIB-3 based electrodes achieves a high optical transparency with 85%, 83%, and 82% transmittance at the visible range, respectively (Fig. 4a). Such a high optical transmittance could be attributed to the transparent nature of SnO_2_, ZnO, and In_2_O_3_, and the high transmittance of the electrolyte and PET substrate. The operating voltage of the symmetrical cell can be widened to 1.2 V without detectable H_2_ or O_2_ evolution after 24 h (Supplementary Fig. 23), which is prerequisite to realize high energy and power densities. The CV curves of MTB-4, MIB-4, and MIB-3 based devices exhibit a nearly rectangular shape at the scan rate of 0.05 V s^−1^ (Fig. 4b), indicating an ideal capacitive behavior and fast electrochemical response. Supplementary Figure 24a–c presents CV curves for MTB-4, MZB-4, and MIB-3 based TFSCs under various scan rates. The lens-like CV shape at scan rates above 0.1 V s^−1^ can be attributed to the unfavorable reaction kinetics under fast charge/discharge rates^95^, which is typical for pseudocapacitive metal oxides^96^. The MTB-4, MZB-4, and MIB-3 symmetric-cell CC test (Fig. 4c) gives a device based areal capacitance (*C*_areal_) of 6.8, 4.9, and 2.7 F cm^−2^ at 0.1 mA cm^−2^, respectively, with excellent rate capability (over 68% capacitance retention when current density increased to 3.2 mA cm^−2^, Fig. 4d). Supplementary Figure 24d shows the variation of *C*_areal_ with respect to scan rates. MTB-4, MZB-4, and MIB-3 based TFSCs exhibit a *C*_areal_ of 5.4, 4.0, and 2.3 F cm^−2^ at a scan rate of 0.03 V s^−1^, and decreases to 4.1, 2.8, and 1.5 F cm^−2^ at 1 V s^−1^, respectively, which demonstrates a similar rate capability as that from galvanostatic charge/discharge test. Considering the combination of energy storage capability and transparency of the TFSCs described here, their performance simultaneously reaches the highest value of *C*_areal_ and transmittance (Fig. 4e). The merit of the TFSCs relative to existing transparent supercapacitors was evaluated using Ragone plot on the device area basis. Building on such optimized capacitive performance and wide operation voltage window, the TFSCs based on MTB-4, MZB-4, and MIB-3 electrode delivered maximum *E*_areal_ of 1.36 × 10^−3^, 9.81 × 10^−4^, and 5.43 × 10^−4^ mWh cm^−2^, respectively, which is superior to the values in recently reported TFSCs^7,65,77–79^ (Fig. 4f).

It is worth mentioning that the active film thickness in MTB, MZB, and MIB electrode in this study was intentionally controlled under 110 nm to maintain the highest transparency. Such a thin active film, however, significantly restricts the areal energy and power density of the TFSCs. To make a fair performance comparison for Fig. 4f, we fabricated MTB-4, MZB-4, and MIB-3 based devices with active film thickness close to the typical non-transparent supercapacitor electrodes. Supplementary Figure 25 shows *C*_areal_ of such non-transparent MTB-4, MZB-4, and MIB-3 supercapacitors (denoted as MTB-4(NT), MZB-4(NT), and MIB-3(NT)) under different active film thicknesses. Clearly, *C*_areal_ significantly increased with the increase of film thickness, which approached to a maximum value of 1102, 732, and 365 mF cm^−2^ for MTB-4(NT), MZB-4(NT), and MIB-3(NT) supercapacitors, respectively. Nevertheless, further increasing the film thickness results in the degradation of *C*_areal_, which may be due to the sluggish ion diffusion across the thicker electrode. Based on such optimized *C*_areal_, MTB-4(NT), MZB-4(NT), and MIB-3(NT) supercapacitors delivered the highest *E*_areal_ of 220, 146, and 73 μWh cm^−2^, respectively. Compared with other non-transparent pseudocapacitive planar systems^42,89,92^, our devices show better performance in terms of areal energy density. Moreover, MTB-4(NT), MZB-4(NT), and MIB-3(NT) supercapacitors also show the maximum areal power density (*P*_areal_) of 36.3, 20.6, and 11.7 mW cm^−2^, 146 and 73 μWh cm^−2^, respectively. These values even compare favorably with other carbon-based planar supercapacitors^88,90,91,93,94^, which normally exhibit high power densities due to the fast charge transfer kinetics^97^.

**Fig. 5:**
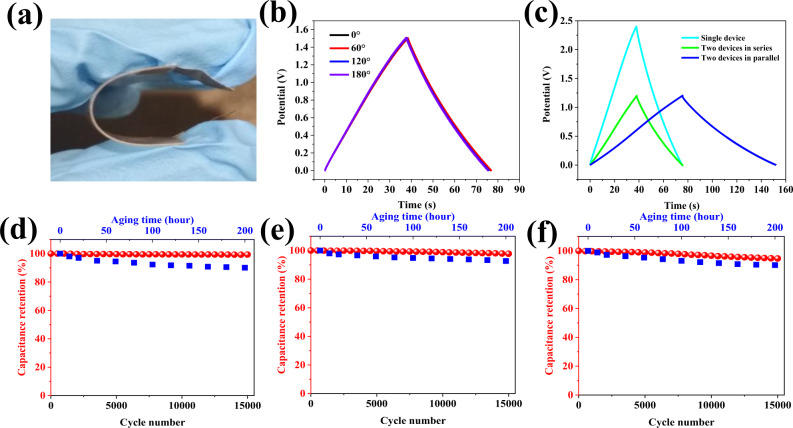
Flexibility, output, durability, and cyclability evaluations of TFSCs. **a** Representative photograph of a fabricated TFSC under bending. **b** CC curves of MTB-4-based TFSCs under various bending angles from 0˚ to 180˚ at 0.1 mA cm^−2^. **c** CC curves of MTB-4-based TFSCs in series or in parallel connection at 0.1 mA cm^−2^. **d**–**f** Capacitance retention of MTB-4, MZB-4, and MIB-3 based TFSCs after 15,000 cycles at 0.05 mA cm^−2^ (under 0–0.8 V voltage window) and 200 aging hours.

The assembled device (5 cm × 4 cm in dimension) was highly robust and flexible (Fig. 5a, based on MTB-4 electrode), being important criteria for the practical application of TFSCs. CC curves showed a symmetric shape and negligible change under various bending angles from 0 to 180˚ (Fig. 5b), indicating an excellent mechanical stability. Moreover, we developed a TFSC stack (Supplementary Fig. 26), which optionally fulfill various output requirements in one device by changing the geometry of connection. For instance, by connecting them in series or in parallel (Supplementary Fig. 26), the device can realize a much-expanded operation voltage (to 2.4 V) or boosted *C*_areal_ (to 9.8 mF cm^−2)^, respectively (Fig. 5c).

Having established the robust redox activities of interstitial boron-doped semiconductor oxides in both electrodes of a symmetric supercapacitor, we further assessed its eligibility for practical applications, starting with their stability in cycled (0-15000 cycles) and aged (0-200 hours) conditions (Fig. 5d–f). Even after 300 h of aging, MTB-4, MZB-4, and MIB-3 based TFSCs still preserve capacitance retention of 91.4%, 88.6%, and 87.7% at 1.2 V, respectively. Moreover, MTB-4-based TFSCs exhibited nearly 100% capacitance retention after 15,000 cycles at 0.05 mA cm^−2^, while MZB-4 and MIB-3-based TFSCs possess a 97% and 95% capacitance retention, respectively. According to above electrochemical characterizations, we believe that the TFSCs in this work show a similar capacitive behavior to previously reported RuO_2_/PEDOT:PSS transparent supercapacitors^12^ (ideal rectangular CV shape) but much better cycling performance: 95–100% capacitance retention after 15,000 cycles in this work vs. 93–100% capacitance retention after 10,000 cycles in RuO_2_/PEDOT:PSS supercapacitors (Supplementary Fig. 27a). Considering other device merits (higher areal capacitance and optical transparency, Supplementary Fig. 27b), we are confident that this work indeed establishes absolute advantages over RuO_2_/PEDOT:PSS-based transparent devices. More importantly, such merits of the TFSCs contain an extendable operating voltage, various optional outputs, good mechanical flexibility, and high durability, thus representing promising candidates to meet the urgent energy storage demand in planar electronics.

## Discussion

In this contribution, we report a family of transparent semiconductor oxides with excellent energy storing capability. This is achieved by facile interstitial boron doping on mesoporous SnO_2_, ZnO, and In_2_O_3_ with tunable pseudocapacitive reactivity. In 1 M KOH aqueous electrolyte, the interstitial boron-doped semiconductor oxides shows a benchmark volumetric capacitance of 1172 F cm^−3^, which enables the most efficient charge storage utilization on ultrathin active film and thus overcomes the trade-off between the transparency and capacitance of the planar electrode. By assembling the two identical transparent electrodes with optimal volumetric capacitance, the obtained flexible supercapacitor exhibits a maximum areal energy density of 1.36 × 10^−3^ mWh cm^−3^, with nearly 100% capacitance retention after 15,000 cycles and ultrahigh transparency (up to 85% transmittance at 550 nm). As a substantial step towards practical application, the fabricated flexible device achieves controllable outputs and excellent durability, which suggests promising opportunities for transparent energy storage in planar electronics.

## Methods

### Preparation of interstitial boron-doped mesoporous semiconductor oxides

Anhydrous SnCl_4_ (98%, Aldrich), Zn(NO_3_)_2_·6H_2_O (98%, Aldrich), In(NO_3_)_3_·5H_2_O (99%, Aldrich) was used as a inorganic precursors for mesoporous samples, and pluronic F127 (Aldrich) was employed as the pore forming agent. Boric acid (Aldrich) was used as precursor of doped B. Deionized water and the ethanol (EtOH, Merck) was used as the solvents. The sol solution for the synthesis of interstitial boron-doped mesoporous semiconductor oxides was prepared by mixing the inorganic precursors, F127, boric acid, deionized water, and the ethanol with the molar ratio of 1:0.0053:(0.05–0.1):18:30 inorganic precursor: F127:H_3_BO_3_:H_2_O:EtOH. The concentration of boric acid in the mixing sol was changed to control the doping level. the obtained sol was stirred under room temperature for 2 h prior to film deposition, and then dip coated on the pre-cleaned glass substrate. The obtained film was firstly aged at room temperature for 2 days under 50% relative humidity (RH). Then the film was annealed at 200 °C for 12 h and calcined at 650 °C under air for 2 h at a ramping rate of 1 °C/min. Finally, the powder scraped from the film on glass substrate was washed with water, filtered, and dried under 60 °C. This procedure was repeated three times to remove the boron oxide formed during the material preparation. Undoped mesoporous samples was prepared in the same method, without the addition of boric acid in the mixing sol.

### Fabrication of transparent electrode based on mesoporous semiconductor oxides

Aerosol-jet spraying was employed to prepare transparent electrode based on mesoporous semiconductor oxides (interstitial boron-doped and pristine). PEDOT:PSS, which was used as conductive agent, was bought from Alfa-Aesar. Fifteen milliliters of 1 mg/ml semiconductor oxides aqueous dispersion was added to the 0.25 mg/ml PEDOT:PSS solution. The liquid was then injected in the syringe that was connected to the spray system. To achieve the most transparent films, the deposition condition, including the nozzle size, sheath gas flow rate, atomizer gas flow rate, was optimized, and all these parameters were summarized in Table S1. The spraying temperature was kept at 110 °C for the fast evaporation of water. To prepare electrode with various thickness, the sprayed volume was kept to 5, 10, 15, and 20 ml.

### Fabrication of mesoporous semiconductor oxide-based electrode using AB, CNT, and KS-6 as a conductive agent

Mesoporous semiconductor oxides (MTB-4, MZB-4, and MIB-3, 86%) were mixed with an NMP solution of PVDF (polyvinylidene fluoride, 7 wt %) and conductive carbons (AB, CNT, or KS-6, 7%) to obtain a slurry. Then the slurry was coated onto graphite foil, which acts as the current collector. After drying at 100 °C, the supercapacitor electrode was obtained. Pristine AB, CNT, and KS-6 electrodes were prepared through a similar method.

### Preparation of substitutional boron-doped semiconductor oxides via LPCVD

LPCVD boron-doped semiconductor oxide layers were deposited on glass substrates. Tetramethyltin, diethylzinc, and metallic indium were employed as precursors for the deposition of SnO_2_, ZnO, and In_2_O_3_, respectively. Diborane (B_2_H_6_) diluted in argon (1%, v/v) was used as a doping agent. The doping ratio (B_2_H_6_/precursor) employed during deposition was fixed to 2. The gas line and mass flow controllers and gas line were modified to permit adequate flow of precursor vapor. The flow rate was kept at 400 sccm and the O_2_ flow rate was kept to 60 sccm during deposition. In this study, the deposition time was held to 20 min and the pressure was controlled at around 4 Torr.

### Structural characterizations

TEM and EELS (Gatan) examinations were performed in a JEOL 2011 microscope. XPS was collected in a PHI-5000C ESCA system (Perkin Elmer) with Mg *K*α radiation (*hν* = 1253.6 eV). X-ray diffraction patterns were conducted on a Brucker D8 powder X-ray diffractometer employing Cu *K*α radiation. Ar adsorption–desorption isotherms were obtained by Quantachrome Autosorb-iQC at 87 K. The specific surface area was calculated based on the quenched solid density functional theory). The pore size distribution was analyzed based on Barrett–Joyner–Halenda method. Optical transmission spectra were recorded in the visible range (350–800 nm) using a Varian Cary 6000i. Sheet resistance of the electrode was measured on the Keithley 2400 source meter. The thicknesses of the micrometer scale thick films were obtained a Dektak 6 M contact profilometry (Veeco Instruments). The resistivity–temperature measurements were performed with a thermal resistance tester (TRT-1000, Wuhan, China).

### Electrochemical characterization

Before electrochemical measurement, transparent electrodes were immersed in a baker containing formic acid to promote the conductivity of PEDOT:PSS. Silver slurry was applied on the edge of the electrode for the connection of external wires. Electrochemical characterizations were performed in several configurations. In three-electrode cells, Hg/HgO and Ag/AgCl was used as the reference electrode when the cell was tested under KOH and H_2_SO_4_ aqueous electrolyte, respectively. CV and galvanostatic charge–discharge (CC) were conducted employing an electrochemical workstation CHI 660E. Electric impedance spectroscopy was collected from 10 mHz to 100 kHz with an amplitude of 10 mV.

### Fabrication of TFSCs

The polyvinyl alcohol/potassium hydroxide (PVA/KOH) gel electrolyte for TFSCs was synthesized as follows: 4 g PVA was firstly mixed with 30 ml deionized water at 80 °C. Meanwhile, 2.4 g KOH was fully dissolved in 10 ml deionized water. Then the two solutions were combined. After vigorous stirring, the transparent gel electrolyte was obtained. To fabricate TFSCs, two identical transparent electrodes were dipped into the above electrolyte for 10 min and dried at 60 °C for 30 min. Afterwards, two electrodes were assembled and dried for 2 h to form a TFSC, where silver paints and aluminum foils were introduced for outward connection.

## Supplementary information

Supplementary Information

## Data Availability

The authors declare that all data supporting the findings of this study are available within the article and Supplementary information files and are also available from the corresponding author upon reasonable request.
