# Author Correction: Interstitial boron-doped mesoporous semiconductor oxides for ultratransparent energy storage

**DOI:** 10.1038/s41467-021-21705-3

**Published:** 2021-03-01

**Authors:** Jian Zhi, Min Zhou, Zhen Zhang, Oliver Reiser, Fuqiang Huang

**Affiliations:** 1 State Key Laboratory of High-Performance Ceramics and Superfine Microstructure, Shanghai Institute of Ceramics, Chinese Academy of Sciences, Shanghai, P. R. China; 2 Institute of Organic Chemistry, University of Regensburg, Universitätsstr. 31, Regensburg, Germany; 3 Hefei National Laboratory for Physical Science at the Microscale, Department of Applied Chemistry, University of Science and Technology of China, Hefei, Anhui P. R. China; 4 SCNU-TUE Joint Lab of Device Integrated Responsive Materials (DIRM), South China Normal University, Guangzhou, China; 5 Beijing National Laboratory for Molecular Sciences and State Key Laboratory of Rare Earth Materials Chemistry and Applications, College of Chemistry and Molecular Engineering, Peking University, Beijing, P. R. China

**Keywords:** Electrochemistry, Supercapacitors, Electrochemistry

Correction to: *Nature Communications* 10.1038/s41467-020-20352-4, published online 19 January 2021.

**Correction 1:** Figure panels 3g and 3h in the original version of this article were inadvertently reversed. Correct Fig. 3 with the updated panels is listed below:

**Fig. 3:**
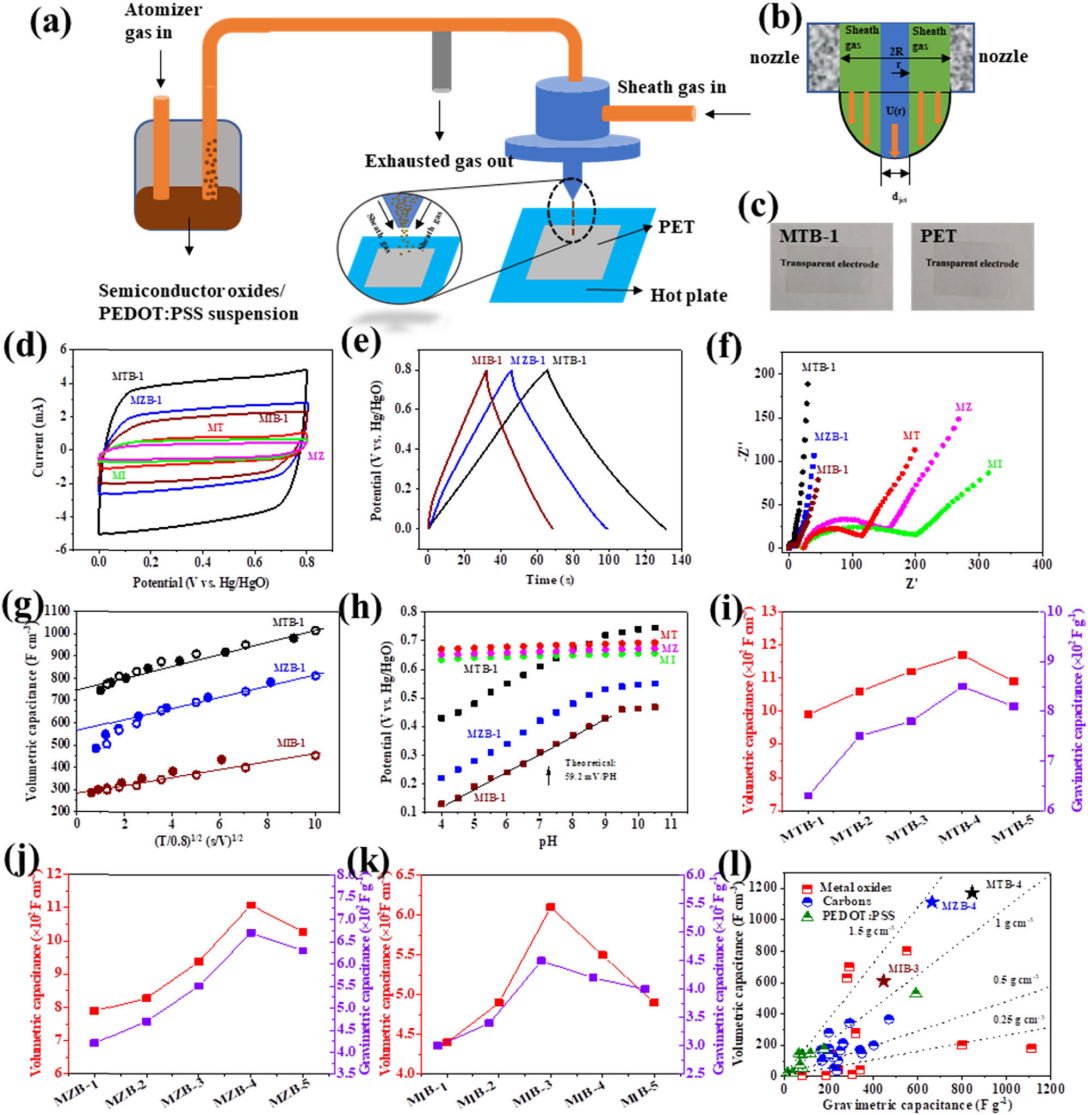
▓.

**Correction 2**: The sentence “we further assessed its eligibility for practical applications, starting with their stability in aged conditions (Fig. 5d–f)” inadvertently omitted some information,the correct sentence should be: “we further assessed its eligibility for practical applications, starting with their stability in cycled (0-15000 cycles) and aged (0-200 hours) conditions (Fig. 5d-f).”

**Correction 3**: The aging condition for MTB-4, MZB-4, and MIB-3 based TFSCs was incorrectly stated as “200 hours”. The correct aging time is “300 hours”. Corresponding sentence should have read “Even after 300 h of aging, MTB-4, MZB-4, and MIB-3 based TFSCs still preserve capacitance retention of 91.4%, 88.6%, and 87.7% at 1.2 V, respectively.”

The error has not been corrected in the PDF or HTML versions of the Article.

