# Supplementary material for: Interstitial boron-doped mesoporous semiconductor oxides for ultratransparent energy storage
Source: Nat Commun. 2021 Jan 19;12:445. doi: 10.1038/s41467-020-20352-4 (PMC7815797; doi:10.1038/s41467-020-20352-4)
Supplement: Supplementary file 1 — Supplementary Information [file 41467_2020_20352_MOESM1_ESM.pdf]

Supplementary Materials for

Interstitial boron-doped mesoporous semiconductor oxides for ultratransparent energy storage

Jian Zhi[1,2], Min Zhou[3], Zhen Zhang[4], Oliver Reiser[2], Fuqiang Huang[1,5]*

[1]State Key Laboratory of High-Performance Ceramics and Superfine Microstructure, Shanghai Institute of Ceramics, Chinese Academy of Sciences, Shanghai 200050, P. R. China.

[2] Institute of Organic Chemistry, University of Regensburg, Universitätsstr. 31, 93053 Regensburg, Germany.

[3] Hefei National Laboratory for Physical Science at the Microscale, Department of Applied Chemistry, University of Science and Technology of China, Hefei, Anhui 230026, P. R. China.

[4] Joint Research Lab of Device Integrated Responsive Materials, South China Normal University, Guangzhou 510631, China.

[5] Beijing National Laboratory for Molecular Sciences and State Key Laboratory of Rare Earth Materials Chemistry and Applications, College of Chemistry and Molecular Engineering, Peking University, Beijing 100871, P. R. China

* Email: huangfq@mail.sic.ac.cn

Supplementary figures

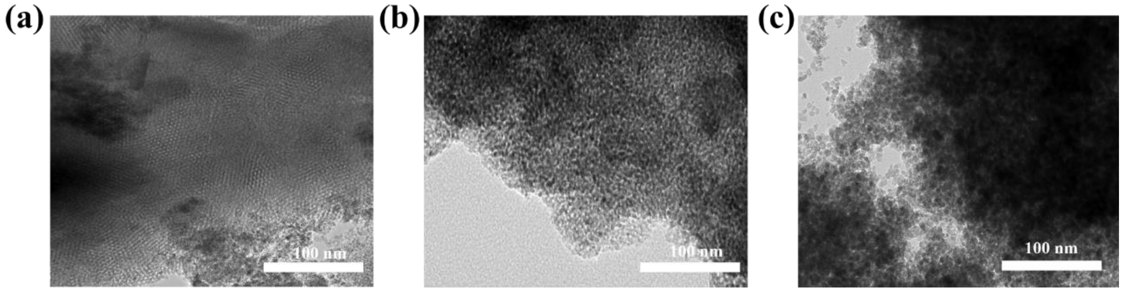

Supplementary Figure 1. TEM images of MT(a), MZ(b) and MI(c).

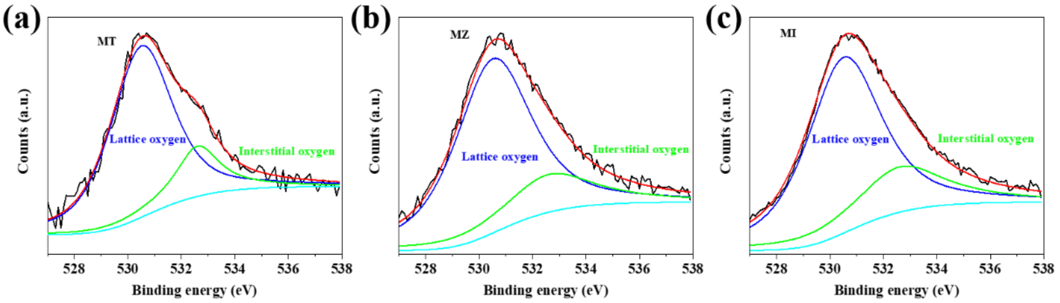

Supplementary Figure 2. High-resolution XPS spectra for O 1*s* of MT(a), MZ(b) and MI(c).

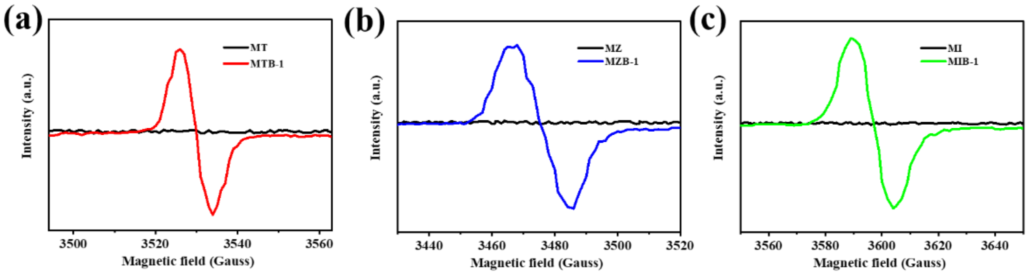

Supplementary Figure 3. EPR spectra of MTB-1(a), MZB-1(b), MIB-1(c) and the undoped MT, MZ and MI(a-c).

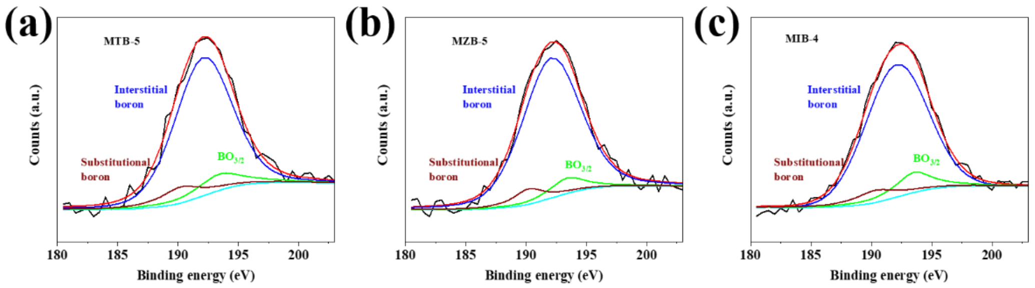

Supplementary Figure 4. High resolution XPS for B 1*s* of MTB-5(a), MZB-5(b) and MTB-4(c).

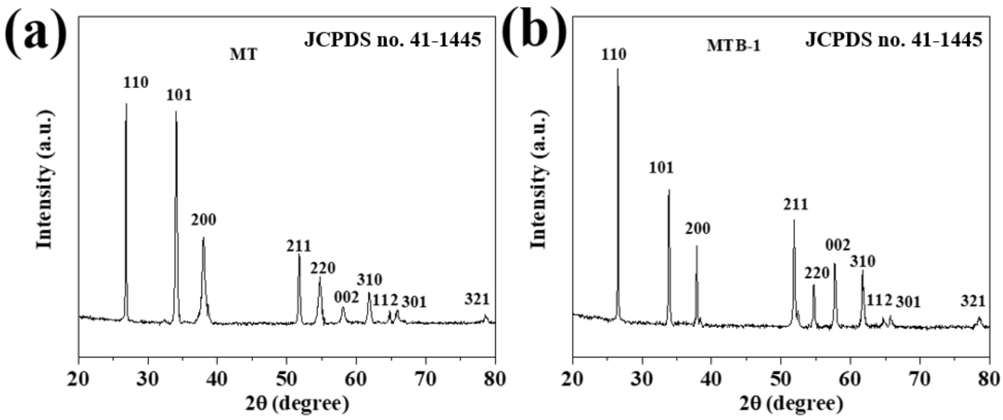

Supplementary Figure 5. XRD of MT(a) and MTB-1(b).

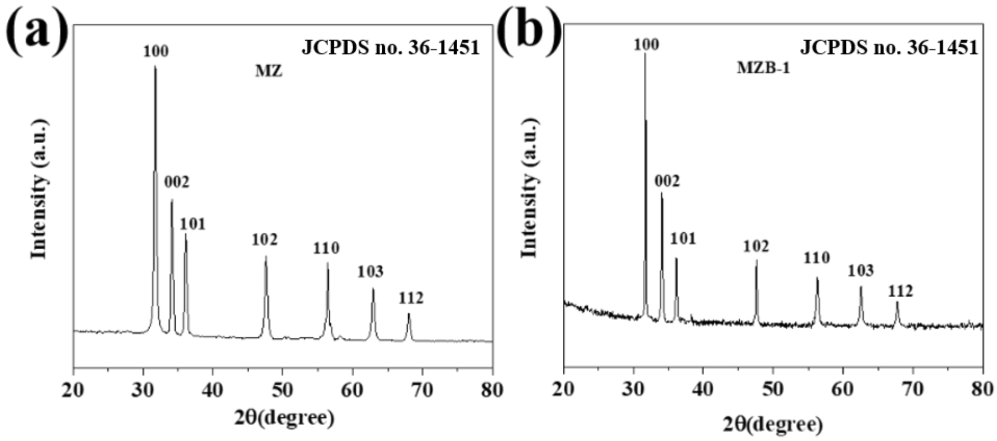

Supplementary Figure 6. XRD of MZ(a) and MZB-1(b).

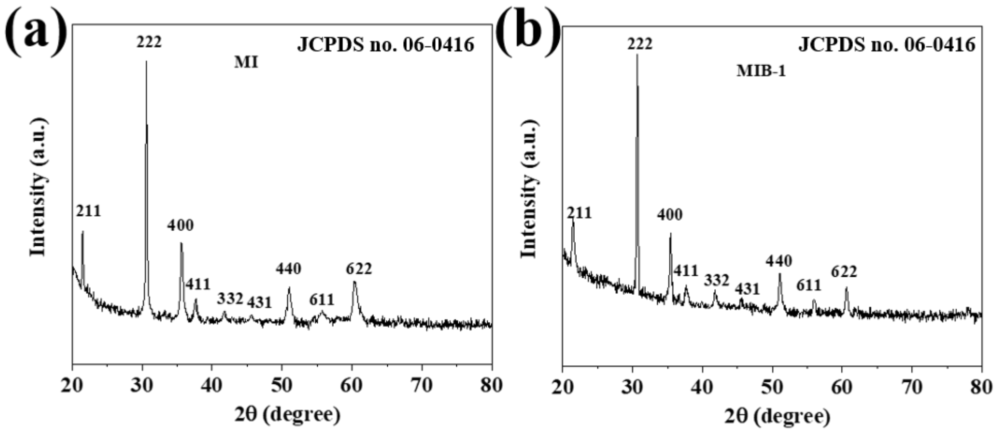

Supplementary Figure 7. XRD of MI(a) and MIB-1(b).

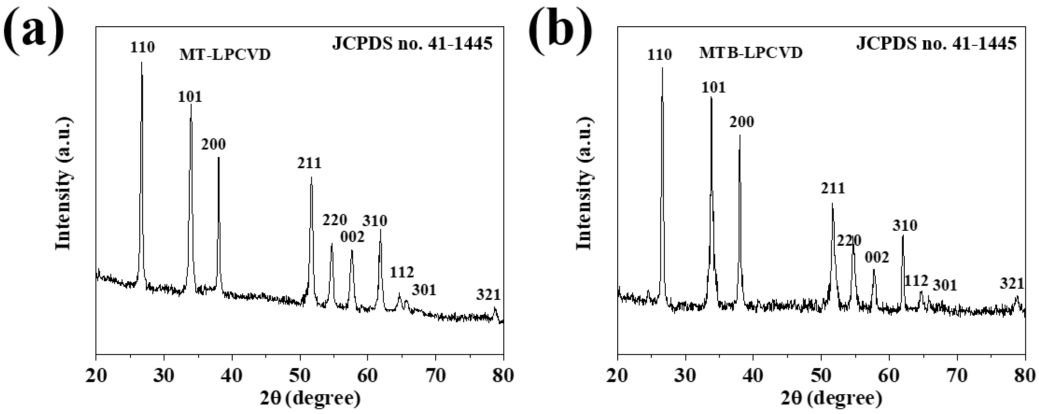

Supplementary Figure 8. XRD of MT-LPCVD(a) and MTB-LPCVD(b).

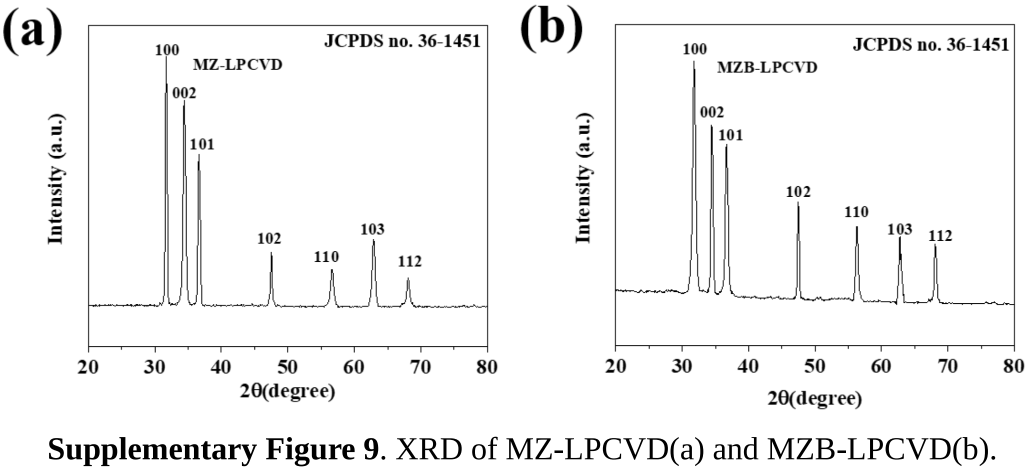

Supplementary Figure 9. XRD of MZ-LPCVD(a) and MZB-LPCVD(b).

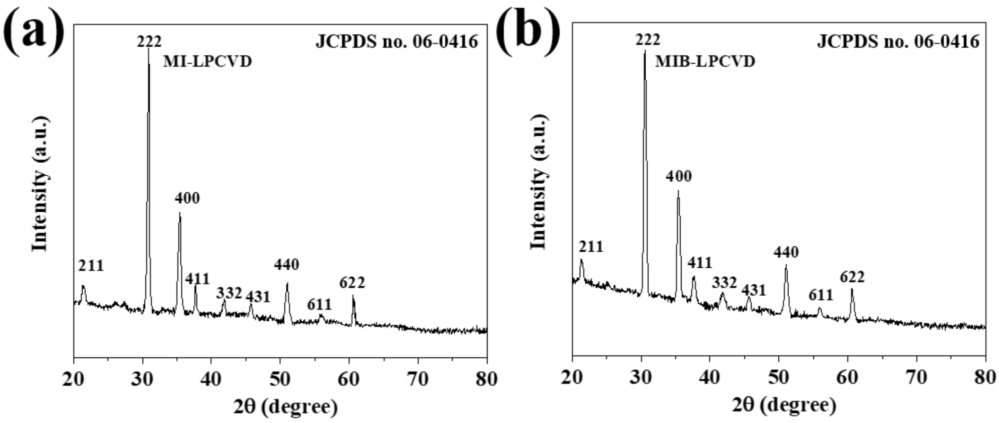

Supplementary Figure 10. XRD of MI-LPCVD(a) and MIB-LPCVD(b).

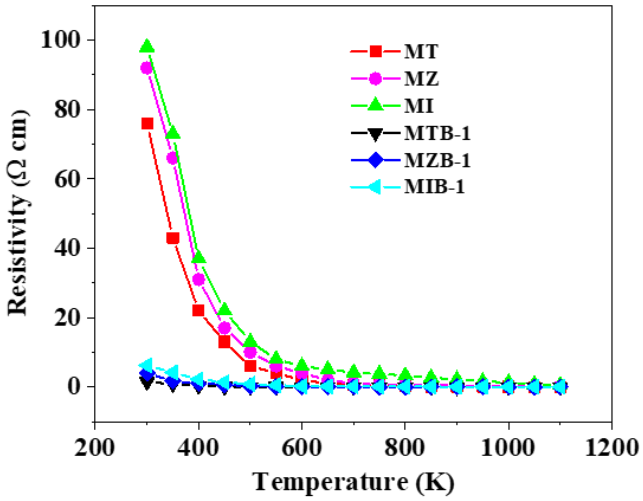

Supplementary Figure 11. The resistivity-temperature curves of MTB-1, MZB-1, MIB-1 and their corresponding pristine samples MT, MZ and MI thin films deposited on silica substrate.

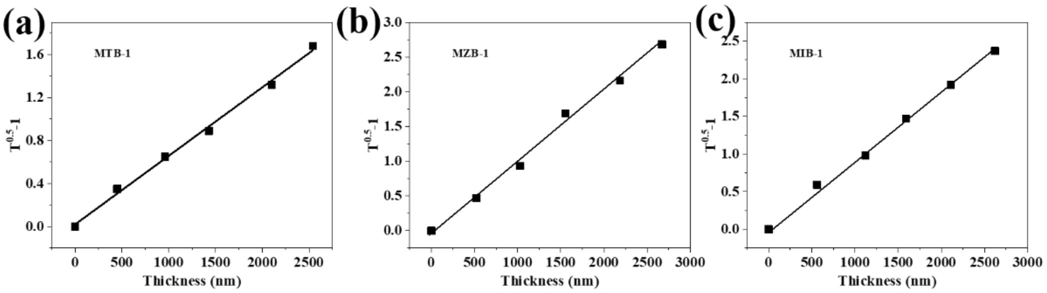

Supplementary Figure 12. Plot of film transmittance express as $T^{-0.5}$–1 as a function of film thickness: (a) MTB-1, (b) MZB-1 and (c) MIB-1.

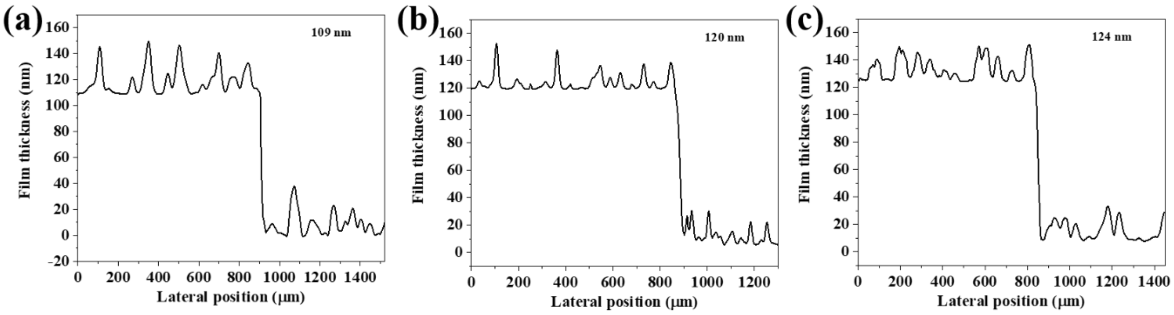

Supplementary Figure 13. (a-c) Representative profilometry results of MTB-4 film at three different testing points. The average thickness of MTB film is 118 nm.

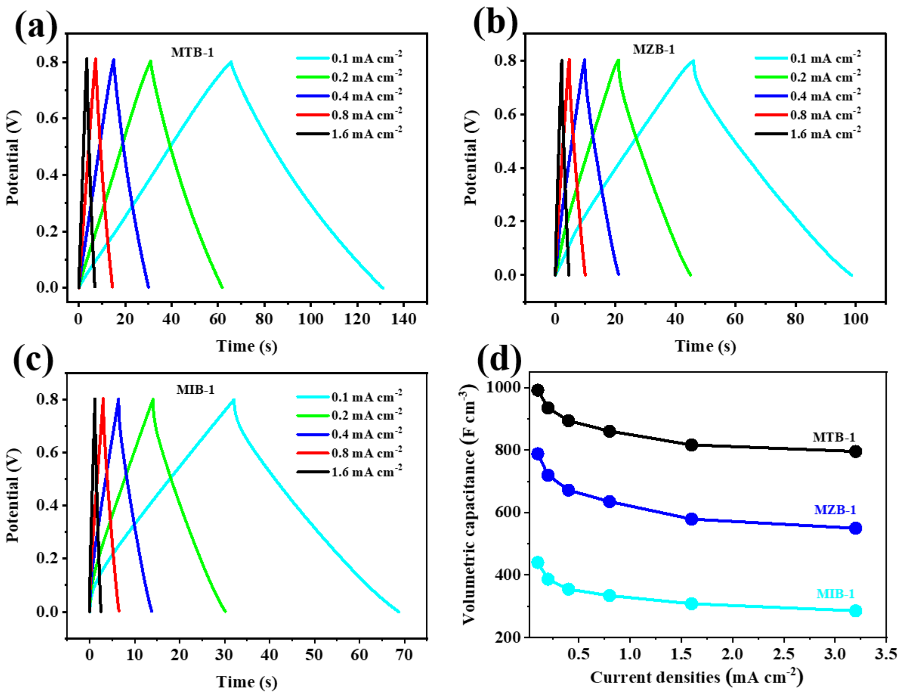

Supplementary Figure 14. (a-c) CC curves of MTB-1, MZB-1 and MIB-1 in various current densities and (d) C_{vol} of MTB-1, MZB-1 and MIB-1 calculated from the CC curves.

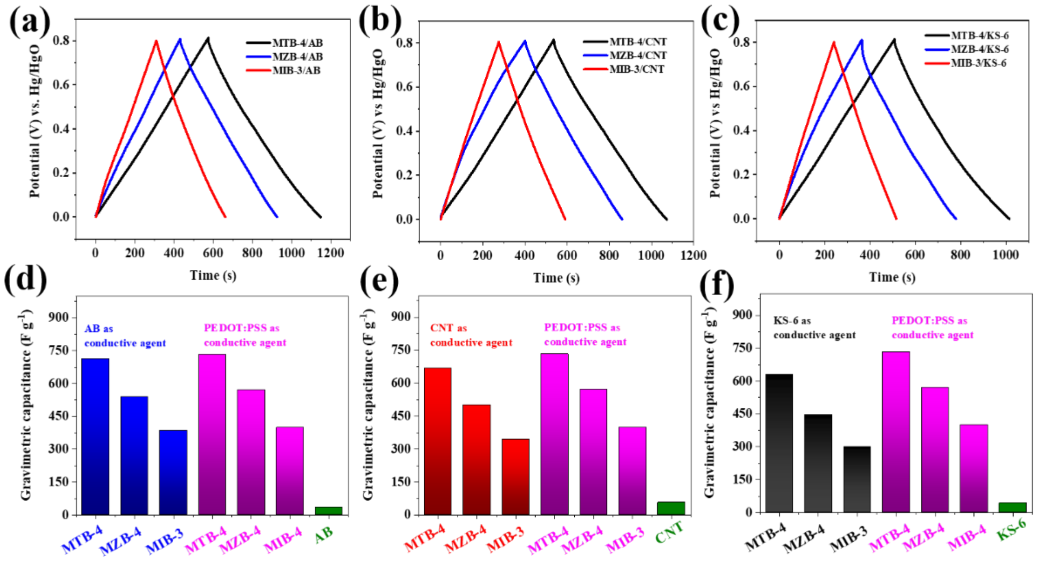

Supplementary Figure 15. (a-c) Galvanostatic charge/discharge profiles of MTB-4, MZB-4 and MIB-3 based non-transparent electrodes at 0.4 mA cm^{-2} under 1M KOH electrolyte, employing AB (a), CNT(b) and KS-6 (c) as conductive agent. (d-f) Corresponding C_{gra} of MTB-4/AB, MZB-4 and MIB-3 based non-transparent electrodes, employing AB (d), CNT(e) and KS-6 (f) as conductive agent at 0.4 mA cm^{-2}. The C_{gra} of MTB-4, MZB-4 and MIB-3 using PEDOT:PSS as conductive agent, as well as the C_{gra} of pristine AB, CNT and KS-6 were also shown in Figure d-f, respectively.

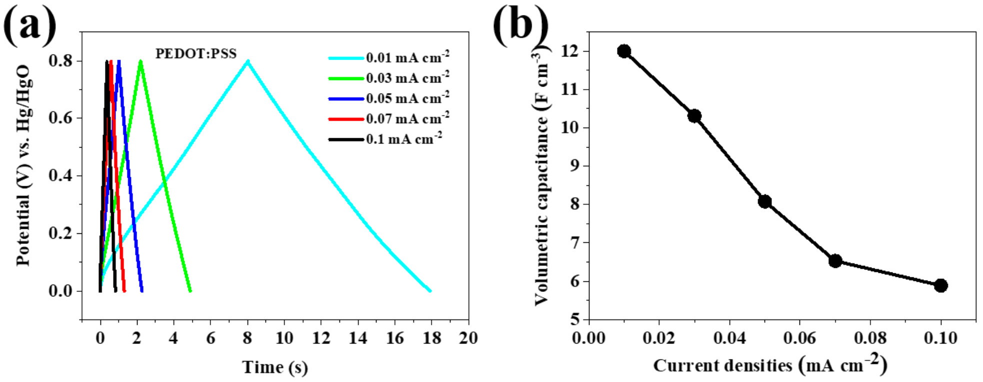

Supplementary Figure 16. Galvanostatic charge/discharge profiles (a) and corresponding C_{vol} (b)of pristine PEDOT:PSS transparent electrode at various current densities.

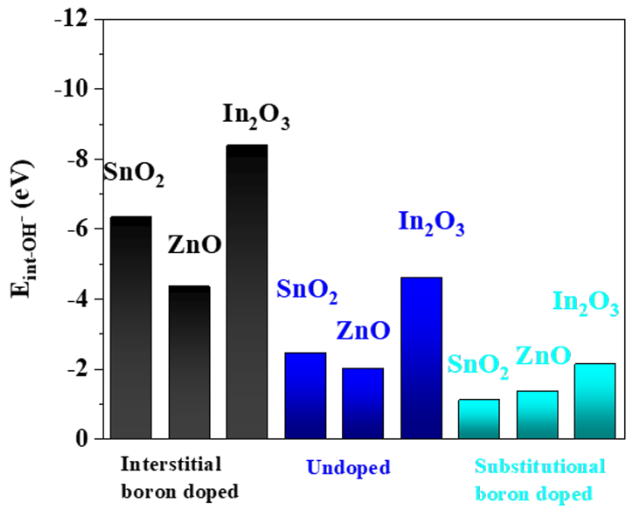

Supplementary Figure 17. OH^- insertion energies of interstitial boron doped, undoped and substitutional boron doped semiconductor oxides

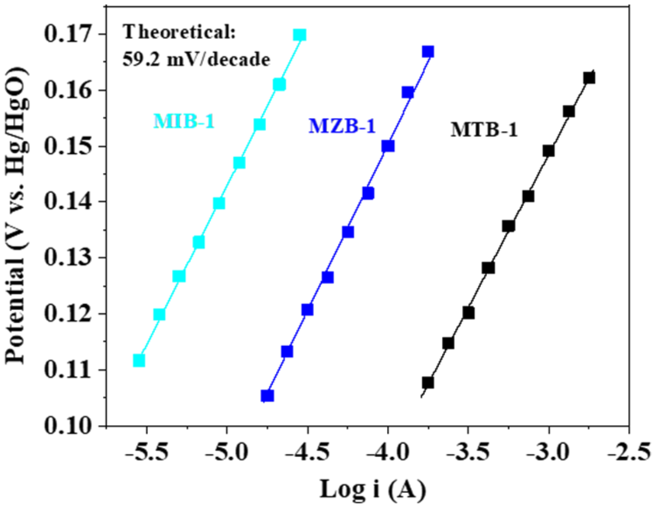

Supplementary Figure 18. Tafel plots of electrode potential against current I in 1M KOH for MTB-1, MZB-1 and MIB-1. All potentials are relative to Hg/HgO reference electrode.

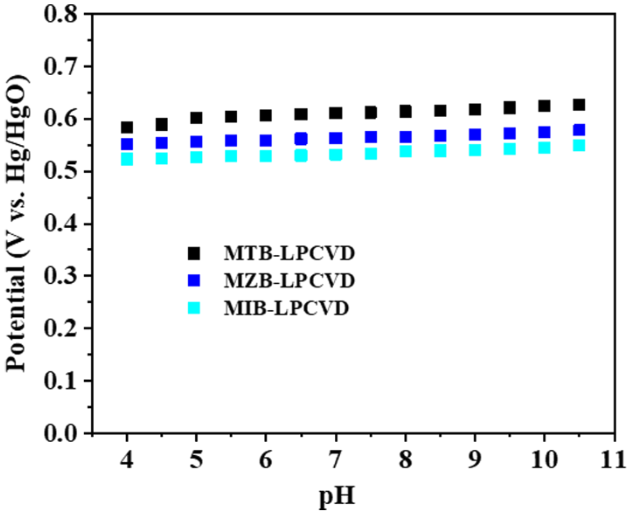

Supplementary Figure 19. Tafel plots of electrode potential against pH value at steady-state current density of 10 mA cm^{-2} for MTB-LPCVD, MZB-LPCVD and MIB-LPCVD.

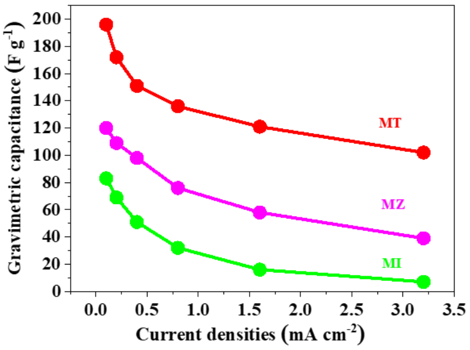

Supplementary Figure 20. C_{gra} of MT, MZ and MI under various current densities.

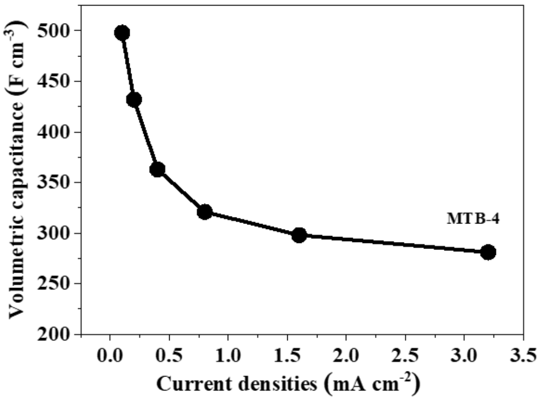

Supplementary Figure 21. C_{vol} of MTB-4 in 1M H_2SO_4 under various current densities.

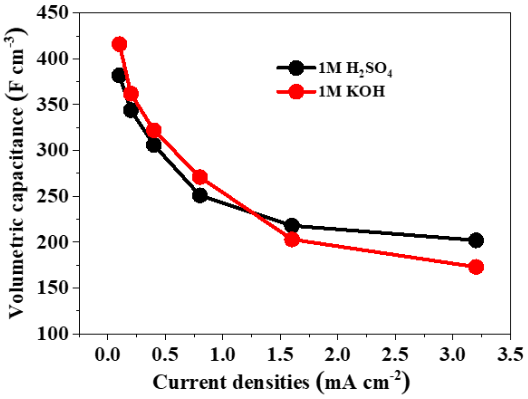

Supplementary Figure 22. C_{vol} of MT in 1M H_2SO_4 and 1M KOH electrolyte under various current densities.

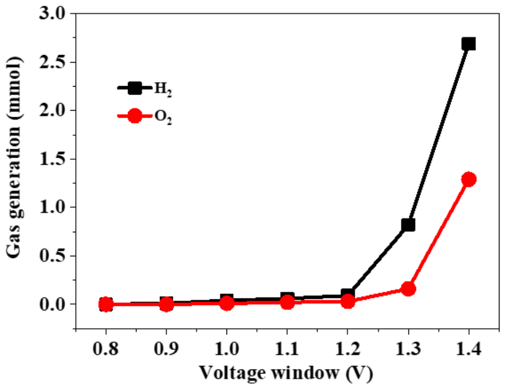

Supplementary Figure 23. Threshold voltage of water splitting determined by H_2 and O_2 accumulation (measured by gas chromatography) in sealed symmetric cell of MTB-4 in 1M KOH electrolyte.

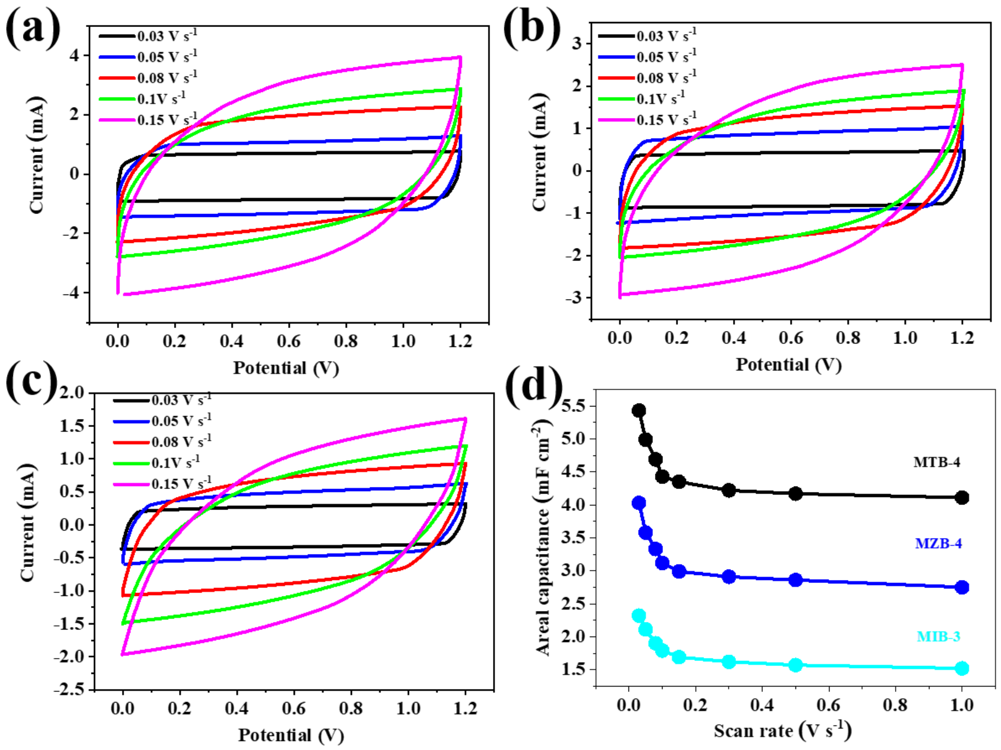

Supplementary Figure 24. (a-c) CV curves for MTB-4, MZB-4 and MIB-4 based TFSCs under various scan rates. (d) Variation of C_{areal} for MTB-4, MZB-4 and MIB-4 based TFSCs with respect to scan rates.

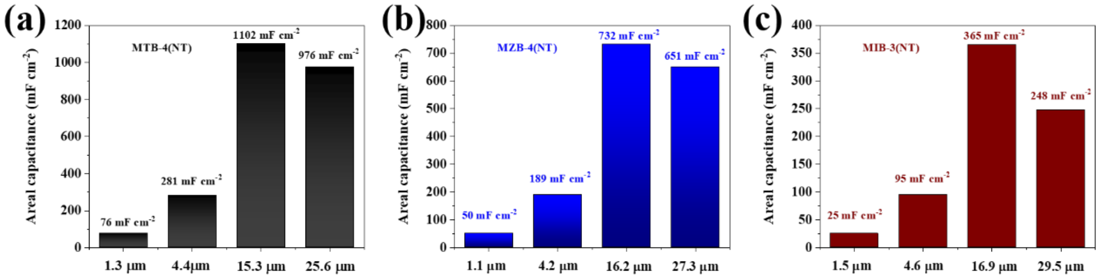

Supplementary Figure 25. Areal capacitance (C_{areal}) of nontransparent MTB-4(a), MZB-4(b) and MIB-3(c) supercapacitors (denoted as MTB-4(NT), MZB-4(NT) and MIB-3(NT)) under different active film thicknesses.

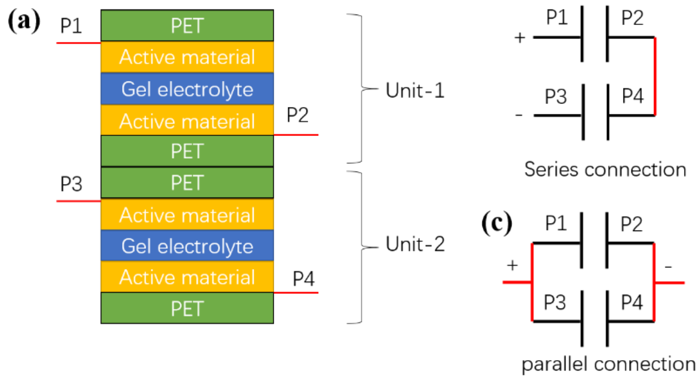

Supplementary Figure 26. (a) Schematic diagram illustrating the design of a smart TFSC stack. (b,c) Circuit diagrams of the connection-dependent outputs by smart ASSC stack.

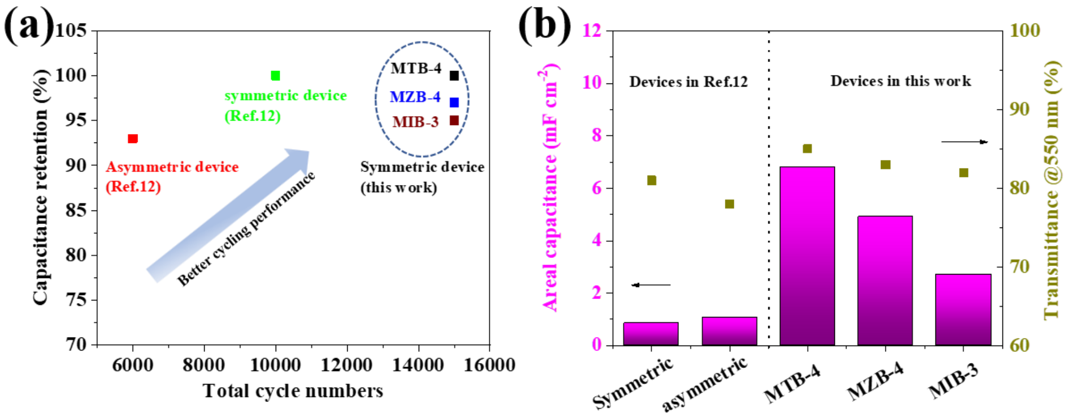

Supplementary Figure 27. Cycling performance (a), areal capacitance and transmittance (b) of TFSCs in this work and RuO_2/PEDOT:PSS transparent devices in Ref. 12.

Supplementary tables

Supplementary Table 1. Boron distribution and porosity parameters obtained from the XPS analysis and nitrogen sorption isotherm for various B-doped samples.

Sample	B(at.%)	%B_{sur}	%B_{ins}	%B_{sub}	S_{BET}(m^2 g^{-1})
MTB-1	3.8	1.4	2.4	0	472
MTB-2	5.4	1.2	4.2	0	436
MTB-3	6.3	0.9	5.4	0	458
MTB-4	7.5	0.7	6.8	0	482
MTB-5	8.1	0.8	6.7	0.6	478
MZB-1	3.5	1.5	2	0	274
MZB-2	4.8	1.4	3.4	0	252
MZB-3	5.6	1.2	4.4	0	293
MZB-4	6.4	1.1	5.3	0	262
MZB-5	6.9	1	5.1	0.8	255
MIB-1	3.1	1.2	1.9	0	156
MIB-2	4.5	0.9	3.6	0	139
MIB-3	5.2	0.7	4.5	0	163
MIB-4	5.9	0.9	4.3	0.7	151
MIB-5	6.4	1	4.1	1.3	160

Supplementary Table 2. Lattice parameters and crystallite size of various semiconductor oxides.

Sample	Lattice parameters				Crystallite size (nm)
	a(Å)	b(Å)	c(Å)	V(Å)^3	
MT	4.802	4.802	3.199	73.77	18.37
MTB-1	4.803	4.803	3.234	74.60	26.81
SnO_2-LPCVD	4.793	4.793	3.186	73.19	13.71
TB-LPCVD	4.766	4.766	3.178	72.19	13.26
MZ	3.250	3.250	5.213	55.06	16.21
MZB-1	3.259	3.259	5.217	55.41	20.38
ZnO-LPCVD	3.253	3.253	5.208	55.11	18.72
ZB-LPCVD	3.250	3.250	3.202	54.95	17.51
MI	10.083	10.083	10.083	1025.11	17.93
MIB-1	10.136	10.136	10.136	1041.36	19.12
In_2O_3-LPCVD	10.153	10.153	10.153	1046.61	14.62
IB-LPCVD	10.151	10.151	10.151	1045.99	14.01

Supplementary Table 3. Deposition conditions and the corresponding film properties based on various MT, MZ and MI samples.

Active material	Deposition conditions			Film properties			
	Nozzle size (μm)	sheath gas flow rate (sccm)	atomizer gas flow rate (sccm)	Sheet resistance (Ω/square)	Transmittance (%)	Optical conductivity (S/cm)	Thickness (nm)[a]
MT	300	280	550	102	91.6	30.6	76(86)
MTB-1	300	280	550	51	91.3	29.2	84(98)
MTB-2	300	280	550	36	90.7	28.7	92(81)
MTB-3	300	280	550	31	90.4	29.1	94(106)
MTB-4	300	280	550	25	90.1	28.1	101(118)
MTB-5	300	280	550	27	89.2	29.8	104(110)
MZ	200	310	530	179	90.7	39.3	68(78)
MZB-1	200	310	530	110	90.2	39.8	71(82)
MZB-2	200	310	530	102	89.6	40.1	75(89)
MZB-3	200	310	530	89	89.8	39.7	78(91)
MZB-4	200	310	530	75	88.9	40.6	79(88)
MZB-5	200	310	530	73	88.6	41.3	81(93)
MI	300	260	510	228	89.5	34.2	88(97)
MIB-1	300	260	510	163	89.1	34.7	91(104)
MIB-2	300	260	510	145	89.2	34.1	92(108)
MIB-3	300	260	510	125	88.7	33.9	97(112)
MIB-4	300	260	510	133	88.3	33.8	101(116)
MIB-5	300	260	510	129	87.8	33.2	107(127)
PEDOT:PSS	300	280	550	118	93.2	24.3	78(99)

[a]The value in the bracket is the average profilometry thickness at three different testing points

Supplementary notes

Capacitance calculation

The per-electrdoe C_{vol} and C_{gra} measured in the three-electrode cell is calculated from the galvanostatic charge–discharge curves according to the eqation (1) and (2):

$$C_{vol} = \frac{It}{Sl\Delta V} \qquad (1)$$

$$C_{gra} = \frac{It}{m\Delta V} \quad (2)$$

where I is the charging current, t is the discharge time, S is the area of the electrode, l and m is the thickness and mass of the active film, and ΔV is the voltage drop opon discharging.

The device based C_{areal} measured in TFSC is calculated from the galvanostatic charge–discharge curves according to the eqation (3):

$$C_{areal} = \frac{It}{S_T \Delta V} \quad (3)$$

where I is the charging current, t is the discharge time, S_T is the total area of the two electrodes, and ΔV is the voltage drop opon discharging.

The areal energy density (E_{areal}) and power density (P_{areal}) of the device are obtained from the following equations:

$$E_{areal} = \frac{1}{2} \times C_{areal} \times \frac{\Delta V^2}{3600} \quad (4)$$

$$P_{areal} = \frac{\Delta V^2}{4 R_{ESR} S_T} \quad (5)$$

where R_{ESR} is the equivalent serial resistance (ESR) of the device. R_{ESR} is obtained from the CC test by dividing the voltage drop (V_{drop}) upon current reversal by twice the value of current, i.e., $R_{ESR}=V_{drop}/2I$.

Computational details

The calculations have been performed including spin polarization using the generalized gradient approximation (GGA) with the PBE functional,[1] and the hybrid B3LYP functional.[2] The Kohn-Sham orbitals were expanded in Gaussian type orbitals (GTO), as implemented in the CRYSTAL06 code. The kinetic energy cutoff was set to 400 eV for the plane-wave basis set. Brillouin zone integration was sampled with the 6×6×6 and 3×3×1 Monkhorst-Pack mesh k-point for bulk and surface calculations, respectively. In all calculations, the forces acting on all atoms are <0.05 eV/Å in fully relaxed structures, and self-consistency accuracy of 5×10^{-5} eV is reached for electronic loops.

The SnO_2 (002) surface was modeled by a periodic four-layer slab repeated in 3×3 surface unit cell with a vacuum region of 20 Å between the slabs along the Z-axis.

The ZnO($10\bar{1}0$) surface was modeled using a 6-layer slab and a p(2 × 2) supercell.

The slab was periodically repeated in the x-y directions with a 1 nm vacuum region between the slabs in the z-direction. In_2O_3 (110) surface was modeled employing a four-layer slab in 1×1 unit cell with the top two layers relaxed in all calculations. We also used a vacuum spacing of 14 Å to avoid interactions between adsorbates and slab images in z direction. For the system of interstitial boron doping, the [BO_3] unit lies in the basal plane of the intestitial cavity. For the system of substitutional boron doping, B atom replaces a three-coordinated O atom in the lattice, and is bound to three metal ions.

The OH^- intercalation energies ($E_{int\text{-}OH^-}$) of a OH^- into the interstitial boron doped, undoped and substitutional boron doped semiconductor oxides (SnO_2, ZnO and In_2O_3) were calculated according to the equation (6):

$$E_{int-OH^-} = E_{semiconductor\ oxides+OH^-} - (E_{semiconductor\ oxides} + E_{OH^-}) \quad (6)$$

where $E_{semiconductor\ oxides+OH^-}$ is the total energy of undoped, interstitial boron

doped or substitutional boron doped semiconductior oxides with one absorbed OH^-; $E_{semiconductor\ oxides}$ is the total energy of undoped, interstitial boron doped or substitutional boron doped semiconductior oxides; E_{OH^-} is the total energy of a OH^- ion.

Supplementary References

1. Hammer, B.; Hansen, L. B.; Nørskov, J. K., Improved adsorption energetics within density-functional theory using revised Perdew-Burke-Ernzerhof functionals. *Physical review B* **1999,** *59* (11), 7413.
2. Finazzi, E.; Di Valentin, C.; Pacchioni, G., Boron-doped anatase TiO2: pure and hybrid DFT calculations. *The Journal of Physical Chemistry C* **2009,** *113* (1), 220-228.
